# From Cages to Sheets: Computational Elucidation of Methylaluminoxane Structure and Reactivity

**DOI:** 10.1002/cplu.70220

**Published:** 2026-08-11

**Authors:** Mikko Linnolahti, Perttu Hanhisalo, Aleksi Vähäkangas

**Affiliations:** ^1^ Department of Chemistry and Sustainable Technology University of Eastern Finland Joensuu Finland

**Keywords:** cocatalyst activation, computational chemistry, density functional theory, methylaluminoxane, olefin polymerization

## Abstract

Methylaluminoxane (MAO) is the most widely used cocatalyst in single‐site olefin polymerization, yet its molecular structures have decisively resisted characterization. Computational chemistry has played an indispensable role throughout the characterization efforts, from the earliest cage‐based structural proposals inspired by *tert*‐butylaluminoxane analogs through systematic hydrolysis‐based modeling to the identification of two‐dimensional sheet structures as the thermodynamically preferred motif. This review traces that computational journey, with particular attention to the methodological advances and failures that shaped it. The discovery that widely used density functional theory methods for MAO carry systematic errors for four‐coordinate aluminum–oxygen environments, and that vibrational entropy rather than electronic energy is the decisive thermodynamic quantity in the experimentally relevant size domain, fundamentally redirected the field. The resulting ovalene‐based sheet model for the dominant [(AlOMe)_16_(AlMe_3_)_6_Me]^−^ anion is simultaneously consistent with electrospray ionization mass spectrometry, solid‐state NMR, X‐ray crystallography, and synchrotron pair distribution function data, and yields catalyst activation barriers in reasonable agreement with experiment. The structural foundation now established for the dominant anionic component of fresh MAO opens a realistic path toward understanding the full complexity of the MAO mixture and toward rational design of next‐generation aluminum‐based cocatalysts.

## Graphical abstract

Methylaluminoxane (MAO), the dominant cocatalyst in metallocene‐based olefin polymerization, has resisted structural characterization for decades. This review traces the computational journey from early cage and nanotube proposals to the identification of two‐dimensional aluminoxane sheets as the thermodynamically preferred motif, and how this structural insight enabled the first quantitative description of catalyst activation.

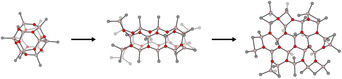

## 1 Introduction

Methylaluminoxane (MAO) is a key cocatalyst in single‐site olefin polymerization, where it activates metallocene and post‐metallocene precatalysts through alkylation and ionization to generate the active cationic species [1, 2]. Since its discovery in the late 1970s by Sinn, Kaminsky, and coworkers [3], MAO has become the dominant cocatalyst in industrial olefin polymerization and the standard activator in academic catalyst development. Remarkably, MAO is the only economical, single‐package cocatalyst known to simultaneously fulfill the roles of alkylator, ionizer, and impurity scavenger. Yet, because MAO is a complex mixture of oligomeric aluminoxane species, its molecular structure has remained one of the most persistently challenging problems in organometallic chemistry. This structural opacity has made rational optimization essentially impossible, and the practical consequences are significant: MAO must be used in large excess, with Al/metal ratios typically in the hundreds to thousands. Further, neither the origin of its exceptional performance nor the identity of its active sites has been established with confidence. These limitations have motivated the search for well‐defined molecular alternatives. A recent striking example is the homodinuclear aluminum cation [iBu_2_(DMA)Al]_2_(μ‐H)^+^, described by Zaccaria, Ehm and coworkers as MAO's “molecular cousin,” which serves as a stand‐alone activator at Al/metal ratios orders of magnitude lower than MAO [4, 5]. The existence of such alternatives highlights both MAO's practical versatility and the cost of its structural complexity: a molecularly defined system can be tuned and optimized in ways that remain out of reach for MAO as long as its structure is unknown.

The difficulty of structural characterization originates from the inherent complexity of MAO: it is not a single compound but a dynamic mixture of oligomeric species. These species form by the controlled hydrolysis of trimethylaluminum (TMA) and can be described with the general formula (AlOMe)_*n*_(AlMe_3_)_*m*_. Size estimates for these oligomeric species range from around 10 to 200 aluminum atoms per molecule. The dynamic equilibria governing MAO in solution have made conventional structural methods exceedingly difficult to apply. NMR spectroscopy describes local chemical environments of aluminum and carbon but cannot resolve individual oligomeric species within the distribution [2]. X‐ray diffraction long remained elusive due to the difficulty of obtaining suitable crystals. This situation changed in 2024, when Luo and coworkers reported the first crystal structure of a MAO component in Science, revealing a two‐dimensional sheet structure for a species with composition (AlOMe)_26_(AlMe_3_)_9_, denoted 26,9 [6].

**FIGURE 1:**
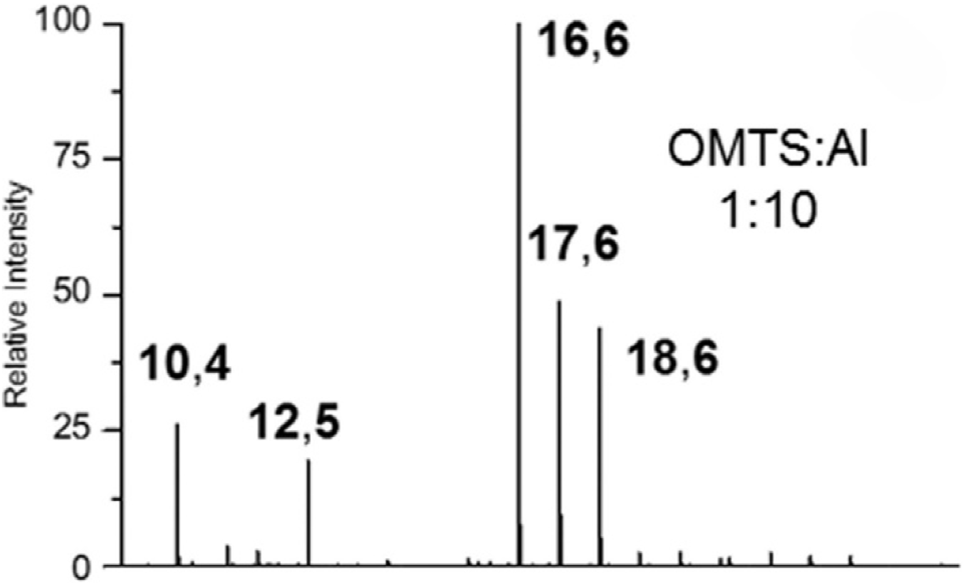
Negative‐ion ESI‐MS spectrum of MAO recorded with OMTS as the donor (OMTS:Al = 1:10), showing the distribution of anionic species of composition (AlOMe)_*n*_(AlMe_3_)_*m*_. The spectrum is dominated by the [16,6]^–^ anion, with minor contributions from neighboring compositions. Adapted with permission [8]. Copyright 2017, American Chemical Society.

Electrospray ionization mass spectrometry (ESI‐MS) has emerged as a particularly powerful tool for probing MAO composition [7]. Upon reaction with neutral donors such as octamethyltrisiloxane (OMTS), MAO forms ion pairs whose anionic components can be detected and characterized by ESI‐MS. It should be noted that ESI‐MS probes species that are both present in solution and amenable to donor‐assisted ionization under the measurement conditions; gas‐phase stability, aging, and sample handling may all influence the observed distribution, and the technique does not directly report the full neutral MAO composition in solution. With this caveat in mind, real‐time monitoring experiments have revealed the evolution of MAO composition during hydrolysis and aging: the anion distribution is consistently dominated by a species designated [16,6]^–^, having the probable composition [(AlOMe)_16_(AlMe_3_)_6_Me]^−^ (Figure 1) [8, 9]. Interestingly, the anions derived from the crystallographically characterized 26,9 [6] are only minor components in these spectra. This observation raises fundamental questions about the relationship between the species that crystallize and those that dominate the mixture.

In the absence of direct structural characterization for most MAO components, computational chemistry has played a central and indispensable role in advancing our understanding. Structural proposals based on chemical intuition and analogy with well‐characterized aluminoxane systems were tested and refined through quantum chemical calculations [10]. The consequences for observable spectroscopic properties were explored through computational NMR, infrared (IR), UV–vis, and X‐ray scattering simulations. These efforts were tightly coupled with experimental observations, particularly ESI‐MS data [11], providing iterative feedback between predicted stabilities and observed species distributions. More recently, systematic benchmarking has revealed significant challenges in the computational treatment of MAO, with widely used density functional theory (DFT) methods showing unexpected biases that necessitate the use of high‐level correlated methods for reliable energetic predictions [12].

In this review, we trace the computational characterization of MAO from early structural proposals and their spectroscopic interrogation through the recent paradigm shift from cage to sheet models. This paradigm shift culminated in the identification of the likely structure of the dominant [16,6]^−^ anion and its role in metallocene catalyst activation. While the emphasis is on computational approaches, we discuss throughout how theoretical predictions have been guided by and validated against key experimental observations, particularly ESI‐MS, NMR spectroscopy, X‐ray crystallography, and X‐ray total scattering, reflecting the inherently interdisciplinary nature of this endeavor. The experimental aspects of MAO synthesis, production, and the influence of preparation conditions on the resulting structural distribution are outside the computational scope of this review and are treated comprehensively elsewhere [2]. The field has reached a point where the central structural questions, long considered intractable, now have well‐founded answers. The remaining challenges are as much about extending and applying this understanding as resolving the structure of MAO itself.

## 2 Structural Proposals

**FIGURE 2:**
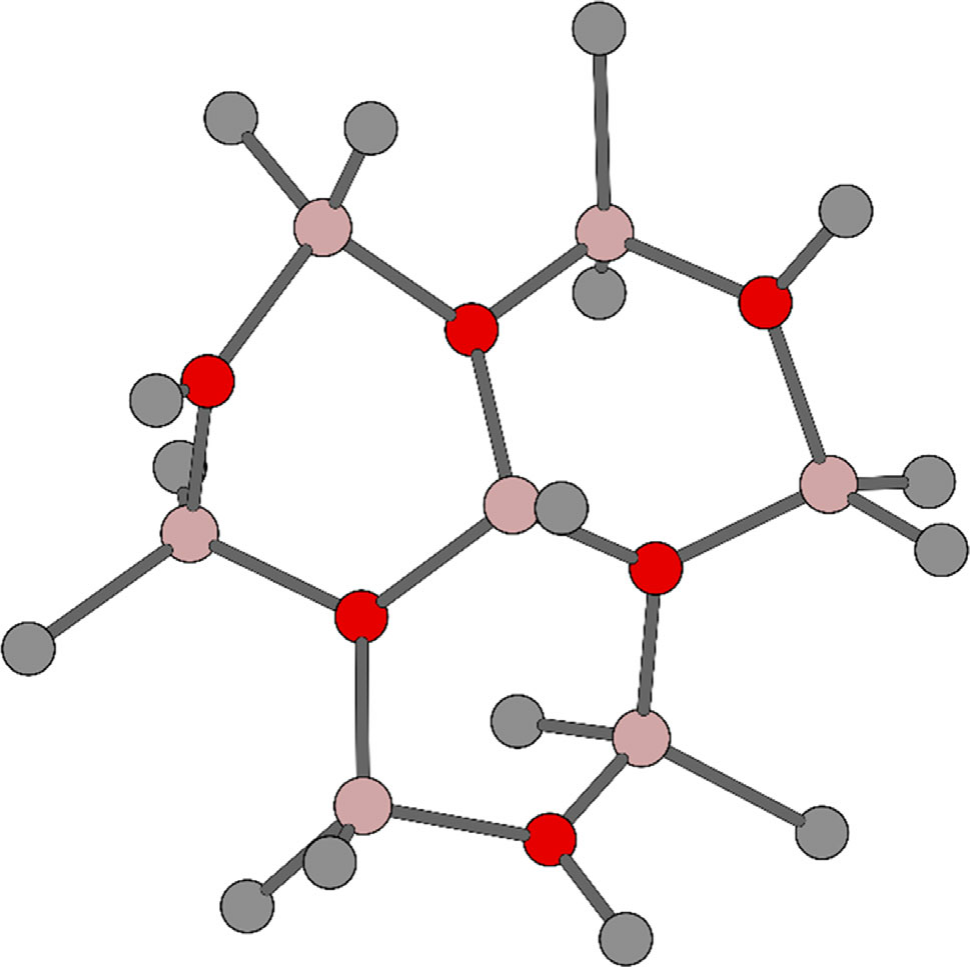
Structure of the Atwood [Al_7_O_6_Me_16_]^−^ anion, the first crystallographically characterized aluminoxane anion. The core is a trihexagon of three fused six‐membered (AlO)_3_ rings with all aluminum atoms in four‐coordinate and all oxygen atoms in three‐coordinate environments. Aluminum atoms are shown in pink, oxygen in red, and carbon in gray. Hydrogen atoms are omitted for clarity.

Structural proposals for MAO have their origin in analogy with small, well‐characterized aluminoxane compounds and in the general principles of aluminum coordination chemistry [13]. The earliest proposals assumed linear or cyclic oligomers with three‐coordinate aluminum and two‐coordinate oxygen atoms. Systematic DFT calculations by Zurek and Ziegler showed that cyclic (AlOMe)_*n*_ structures reach asymptotic stability with increasing ring size but are substantially less stable than three‐dimensional alternatives [14]. The experimental evidence pointing toward higher‐coordinate aluminum in aluminoxane chemistry came from Atwood and coworkers in 1983, who crystallographically characterized the [Al_7_O_6_Me_16_]^−^ anion (Figure 2) [15]. The core of this structure is a puckered polyhexagon of three fused six‐membered (AlO)_3_ rings (a trihexagon) in which all aluminum atoms are four‐coordinate, and all oxygen atoms are three‐coordinate, making it topologically a fragment of a two‐dimensional sheet. Although the [Al_7_O_6_Me_16_]^−^ anion does not conform to the (AlOMe)_*n*_(AlMe_3_)_*m*_ composition characteristic of MAO, and its parent neutral has not been identified, this early result established that four‐coordinate aluminum and three‐coordinate oxygen are the preferred coordination environments in aluminoxane frameworks, a conclusion that would be vindicated four decades later by the first crystal structure of a MAO component [6].

**FIGURE 3:**
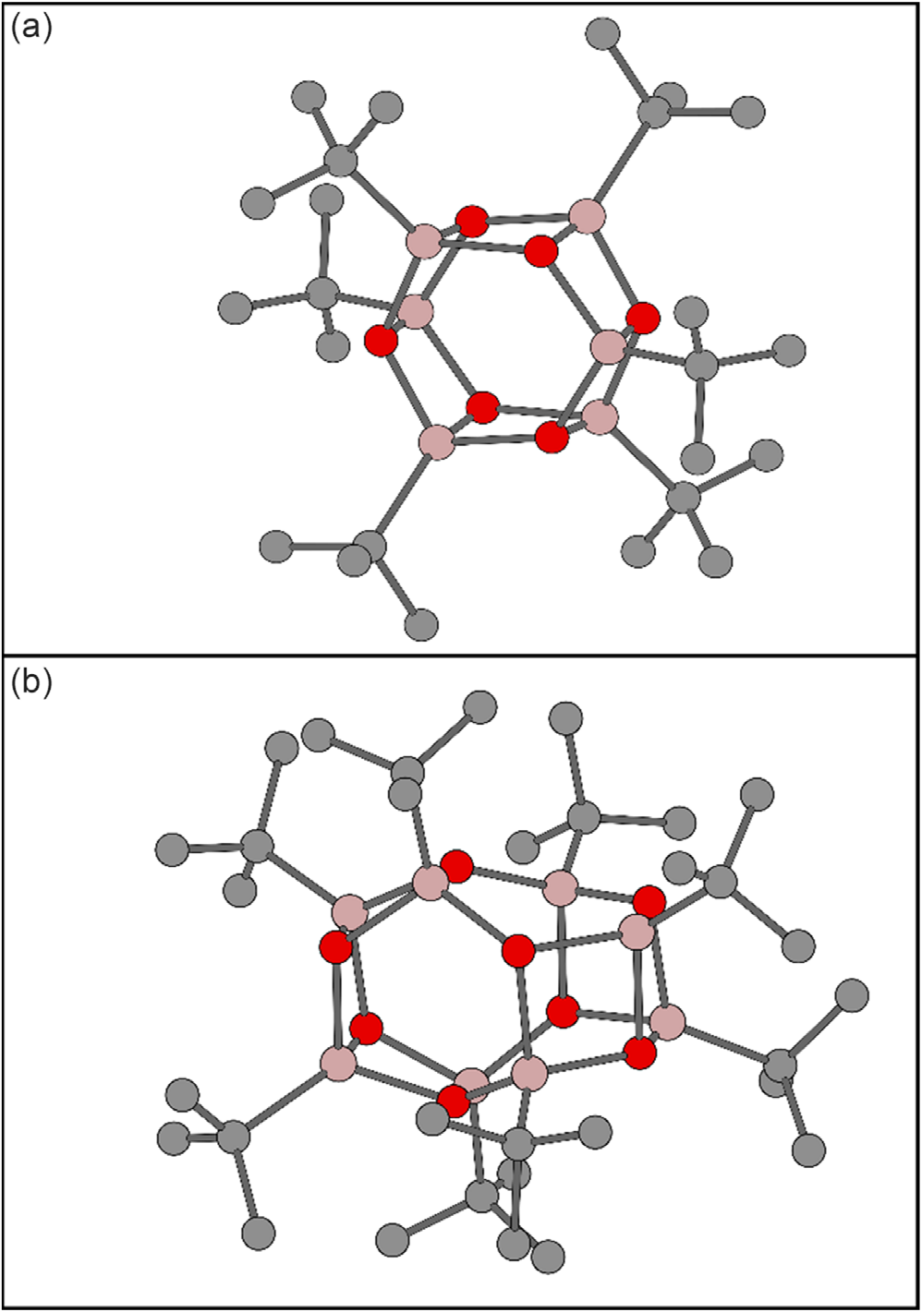
Structures of two *tert*‐butylaluminoxane cages [(*^t^* Bu)Al(μ_3_‐O)]_*n*_ synthesized and crystallographically characterized by Barron and coworkers. (a) *n* = 6, (b) *n* = 8. Aluminum atoms are shown in pink, oxygen in red, and carbon in gray. Hydrogen atoms are omitted for clarity.

The cage paradigm for MAO was largely shaped by the landmark work of Barron and coworkers [16, 17], who synthesized and crystallographically characterized a series of *tert*‐butylaluminoxane cages, [(*^t^* Bu)Al(μ_3_‐O)]_*n*_ (*n* = 6–9 and 12) (Figure 3). These structures consist exclusively of four‐ and six‐membered rings (square and hexagonal faces), obeying the topological relationship S = O + 6. This relationship connects the number of square (S) and octagonal (O) faces, reducing to exactly six square faces when only square and hexagonal faces are present (Smith's rule). The ring strain that is concentrated at the {Al_2_O_2_} square faces provided the basis for Barron's concept of latent Lewis acidity (LLA). This concept states that the most strained sites are also the most reactive toward Lewis bases, including the aluminum alkyls that associate with MAO.

The earliest computational efforts focused not on the global structure of MAO but on the electronic character of its aluminum sites. Yamasaki applied ab initio and density functional calculations to model aluminoxane fragments, evaluating the relative Lewis acidity of different aluminum sites through chloride binding energies and examining the ligand exchange process with a group IV metallocene [18]. Fusco and coworkers studied the reactions of Cp_2_MtMeCl (Mt = Ti, Zr) with TMA and an oxygen‐bridged MAO model by DFT. Their work gave an account for the higher cocatalytic activity of MAO relative to TMA through the energetics of cation formation, noting that tricoordinate aluminum bridging dicoordinate oxygen is expected to be less Lewis acidic than monomeric TMA [19]. Zakharov and coworkers extended these site‐level studies to a series of (AlOMe)_*n*_ cage models (*n* = 4–12), finding that three‐dimensional cage structures become more stable than cyclic alternatives from *n* = 6 onward, that the Lewis acidity of aluminum centers increases with cage size, and that TMA association occurs through simultaneous coordination to Lewis acidic aluminum and Lewis basic oxygen sites [20]. These studies established the conceptual vocabulary of Lewis acidic and basic sites that would underpin much of the subsequent structural debate.

**FIGURE 4:**
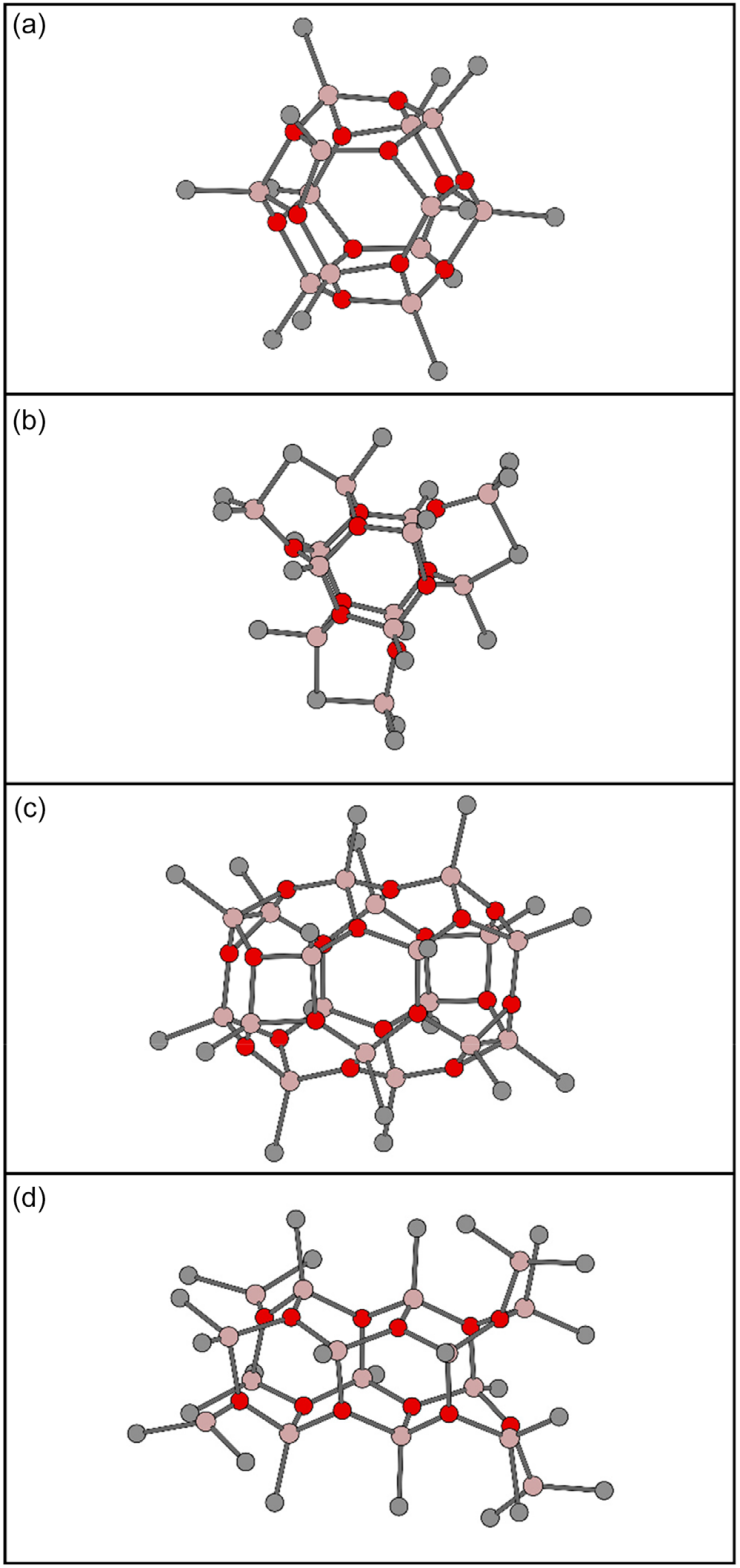
Structural proposals for methylaluminoxane prior to the identification of sheet structures: (a) truncated octahedral (AlOMe)_12_ cage (Zurek and Ziegler), (b) Me_18_Al_12_O_9_ cage (Ystenes and coworkers), (c) (AlOMe)_18_ cage (Boudene and coworkers), (d) armchair nanotube (Linnolahti and coworkers). Aluminum atoms are shown in pink, oxygen in red, and carbon in gray. Hydrogen atoms are omitted for clarity.

Zurek and Ziegler undertook the first comprehensive computational survey of (AlOMe)_*n*_ cage structures, evaluating the energies of 36 different oligomers (*n* = 4–16) using DFT [14]. They found that the stability of individual aluminum sites follows the ordering 3H > 2H + S > 2S + H > 3S, where H and S denote hexagonal and square faces sharing a given vertex. This analysis favored “spherical” cages that minimize the number of destabilizing adjacent square faces. Boltzmann‐weighted Gibbs free energy distributions predicted (AlOMe)_12_ as the most abundant species across a wide temperature range (Figure 4). This cage adopts the topology of a truncated octahedron, the Archimedean solid obtained by cutting the vertices of a regular octahedron, consisting of eight hexagonal and six square faces with no shared square–square edges, making it the smallest (AlOMe)_*n*_ cage in which the particularly destabilizing fused {Al_2_O_2_} squares can be entirely avoided. The same topology is found in Barron's crystallographically characterized (AlO^t^Bu)_12_ complex [16].

Experimentally, however, the ^1^H NMR method developed by Imhoff and coworkers established that the number of methyl groups per aluminum in MAO (excluding free TMA) is consistently in the range of 1.4–1.5, with an Al:O ratio of approximately 1:0.8 [21]. This ratio deviates substantially from the 1:1:1 stoichiometry of the (AlOMe)_*n*_ formula, indicating that significant amounts of TMA are incorporated into the aluminoxane framework. This compositional discrepancy gave rise to an early and useful distinction between two idealized descriptions of MAO. The pure (AlOMe)_*n*_ stoichiometry, sometimes termed “classic” MAO, corresponds to fully condensed aluminoxane built exclusively from [AlOMe] units. The composition actually observed, however, demanded the incorporation of additional TMA, leading Sinn to propose the associated formula Al_12_O_12_(CH_3_)_12_⋅[Al(CH_3_)_3_]_4_, termed “true” MAO [22, 23]. In the *n*,*m* notation used throughout this review, this corresponds to a 12,4 composition, and the general associated formula (AlOMe)_*n*_(AlMe_3_)_*m*_ that frames the modern structural picture is a direct generalization of this idea. As a consequence, simple (AlOMe)_*n*_ cages alone cannot account for the composition of MAO. In a combined experimental and computational study, Ystenes, Rytter, and coworkers proposed the general formula Me_6*m*_Al_3*m*_O_3*m*_ for MAO. They identified Me_18_Al_12_O_9_ cages (*m* = 3) as the most plausible candidates (Figure 4) based on in situ FTIR spectroscopy, chemical analysis, and DFT calculations [24]. A central finding was that TMA incorporation proceeds by opening {Al_2_O_2_} square faces to create Al–Me–Al bridging methyl groups situated in six‐membered rings, and that these bridges are essential for metallocene activation. Rytter et al. further argued that the concept of LLA is misleading: heterolytic Al–O cleavage generates not only a Lewis acidic aluminum but also a highly basic oxygen, and the true driving force for TMA association is bond formation between this basic oxygen and the incoming TMA.

Boudene and coworkers later revisited (AlOMe)_*n*_ cages using meta‐GGA functionals (M06) and a larger set of 56 optimized structures (*n* = 6–12) [25]. They introduced a quantitative geometric descriptor, the average angular distortion of aluminum sites. This descriptor correlated strongly with the normalized electronic energy and provided a general framework for predicting thermodynamic stability regardless of the specific face types present. Extrapolation to larger systems using [AlHO]_*n*_ surrogates, an approach introduced earlier by Earley to calculate the relative stabilities of aluminoxane rings and cages and to compare their simulated IR spectra with experimental MAO [26], predicted a window of thermodynamically stable cage sizes between *n* = 12 and 24, with (AlOMe)_18_ as the most abundant species at room temperature (Figure 4). Smaller oligomers were disfavored by ring strain, while larger cages were destabilized by entropic effects at elevated temperatures.

An alternative to the cage model was proposed in 2006 by Linnolahti, Severn, and Pakkanen, who used periodic ab initio calculations to demonstrate that aluminoxane nanotubes (Figure 4), analogous in construction to carbon nanotubes, are energetically preferred over cages for MAOs [27]. The preference was found to be substituent‐dependent: MAO nanotubes are favored over the truncated‐octahedral (AlOMe)_12_ cage by almost 20 kJ mol^–1^ per {AlO(Me)} unit, whereas for *tert*‐butyl substituents the analogous cage is strongly preferred due to steric overcrowding in the tubular geometry. This result carried an implication beyond the nanotube proposal itself: the experimentally characterized *tert*‐butylaluminoxane cages are poor structural models for MAO, as the substituent profoundly influences the preferred topology. Further computational work elaborated on the nanotube model by exploring capping of finite tubes with TMA and examining growth mechanisms by which cage dimerization spontaneously produces nanotubular segments [28].

Despite these advances in modeling both the (AlOMe)_*n*_ core and its interaction with associated TMA, the structural proposals remained largely guided by chemical intuition and analogy, and direct experimental validation of any specific model proved elusive. The spectroscopic and scattering methods available to interrogate these proposals are the subject of the following subsection. Afterward, the discussion returns to the structural question in the context of synthesis‐based approaches and the eventual emergence of the sheet model.

## 3 Computational Spectroscopic Characterization

### 3.1 Computational NMR Spectroscopy

Among spectroscopic techniques, ^27^Al NMR is the most natural probe of MAO structure owing to its sensitivity to local coordination geometry and its wide chemical shift range. This range spans from near 0 ppm for highly symmetric six‐coordinate environments to above 150 ppm for three‐coordinate aluminum. However, ^27^Al is a quadrupolar nucleus (*I* = 5/2) with large quadrupole coupling constants in MAO. The resulting requirement for simultaneously high magnetic fields and fast magic‐angle spinning rates made reliable solid‐state spectra of pristine MAO inaccessible for many years. Computational prediction of electric field gradient tensors and chemical shifts has therefore played an indispensable role throughout, not merely as an interpretive complement to experiment but as a prerequisite for understanding what the experimental spectra actually contain.

The experimental foundation for understanding the ^27^Al NMR of MAO was laid by Babushkin, Talsi, and coworkers, whose multinuclear (^27^Al, ^1^H, ^17^O, ^13^C) study established that the ^27^Al resonance of MAO is extremely broad at room temperature and sharpens only at elevated temperatures, a direct manifestation of the large quadrupolar coupling and structural heterogeneity that would challenge all subsequent characterization [29]. Bryant, Hall, and coworkers reported the first systematic combined solid‐state ^27^Al NMR and ab initio study. They calibrated EFG tensor calculations against discrete aminato‐ and propanolato‐aluminum cluster compounds before applying them to MAO models. Their results established an upper bound for the quadrupole coupling constant in MAO of approximately 22 MHz. They also showed that a simple hexameric cage structure failed to reproduce the experimental linewidths of MAO gel, while edge‐linked and face‐linked cage dimers performed considerably better. Equally important was the practical conclusion that even the then state‐of‐the‐art 19.6 T/35 kHz MAS conditions were insufficient to acquire an undistorted spectrum of pristine MAO, setting quantitative instrumental requirements for all subsequent work [30].

In parallel, Zurek and Ziegler addressed the solution‐phase ^1^H and ^13^C NMR signatures of metallocene/MAO adducts using cage‐based MAO models. They successfully identified µ‐Me bridging between aluminum and zirconium as a structural feature of the active species, achieving reasonable agreement with experimental chemical shifts [31]. This finding represented an early computational success in connecting MAO structural models to observable spectroscopic signatures, with the µ‐Me bridging motif proving durable in subsequent work [31].

Subsequent computational studies clarified how the aluminum coordination environment controls ^27^Al chemical shifts. Wu and coworkers demonstrated good agreement between computed parameters for a methylated isobutyl aluminoxane and its experimental counterpart, validating the theoretical methodology for four‐coordinate aluminum environments [32]. Boudene and coworkers then showed that aluminum atoms in six‐membered rings are shielded relative to those in four‐membered rings, providing a geometry‐based interpretation framework for the broad, overlapping resonances seen experimentally [25].

A significant methodological advance came from Falls, Zurek, and Autschbach, who for the first time simulated full Boltzmann‐weighted ^27^Al MAS solid‐state NMR spectra of MAO at variable temperature and magnetic field strength. Rather than computing the spectrum of a single model compound, they calculated ^27^Al NMR shielding and quadrupolar coupling tensors for all species in the temperature‐dependent distribution derived from Zurek's earlier cage‐based thermodynamic study. These results were then combined into population‐weighted simulated spectra at 298 K and 19.6 T, giving reasonable agreement with available experimental data for MAO gel, representing the most realistic simulation attempted to that point. A practically important prediction emerged: the ratio of three‐coordinate to four‐coordinate aluminum should be quantifiable from variable‐temperature and variable‐field measurements. The ratio of these atoms relates directly to bound TMA content and therefore to the number of potentially active sites. The differentiation of three‐coordinate and four‐coordinate Al atoms is possible because they contribute characteristically different spectral features. The limitation of this study, acknowledged by the authors, was that the structural distribution consisted entirely of cage‐based models, meaning the simulated spectra reflected the best available structural hypothesis rather than established ground truth [33].

The first NMR study to use a sheet model for MAO came from Collins and Linnolahti, who computed ^13^C chemical shifts for metallocene/MAO adducts formed with the 16,6 sheet model (described further in the Sheet Structures section). They found agreement with experimental solution‐phase spectra comparable to that obtained with cage‐based models. This result was instructive in a negative sense: NMR chemical shifts in these adducts are dominated by the local coordination environment around zirconium rather than the extended structure of the counter‐anion. Therefore, ^1^H and ^13^C solution NMR of metallocene/MAO mixtures cannot readily discriminate between sheet and cage counter‐anion structures. Structural assignment requires either solid‐state techniques sensitive to the aluminum framework itself, or methods such as ESI‐MS that directly report on MAO composition [34].

**FIGURE 5:**
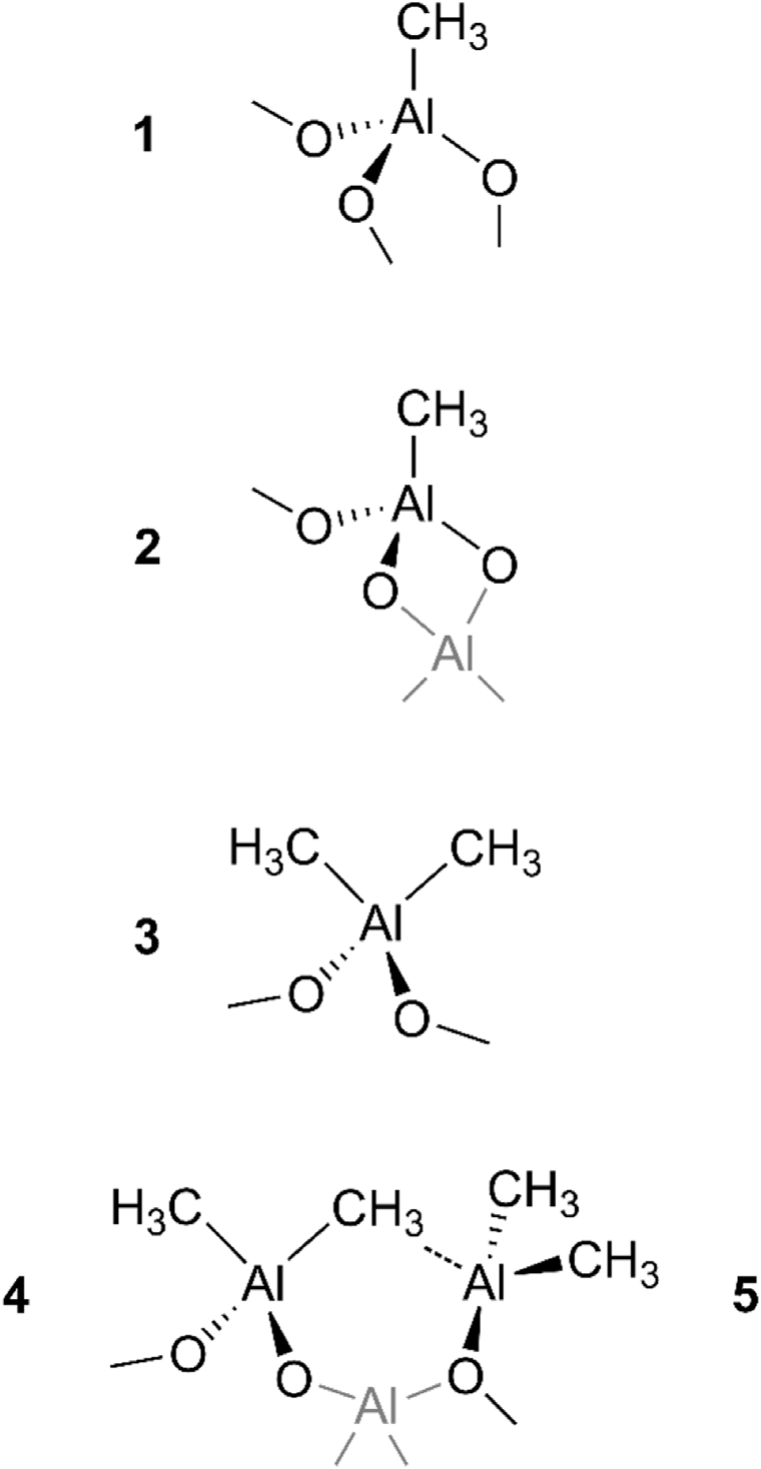
The five distinct aluminum environments in MAO resolved by ^27^Al solid‐state NMR at ultrahigh field. **1** and **2** are [AlO_3_Me] backbone environments common to both cage and sheet structures; **3** and **4** are bismethyl aluminum environments in which a TMA‐derived AlMe_2_ group is stabilized by a bridging methyl from a neighboring aluminum center; **5** is the Lewis acidic three‐coordinate site whose intensity decreases upon treatment with Lewis bases.

The most powerful NMR study of MAO to date was reported by Taoufik, Autschbach, Delevoye, Gauvin, and coworkers. They exploited an ultrahigh magnetic field of 28.2 T combined with the DFS‐coslp‐MQMAS pulse sequence specifically designed to enhance excitation of sites with large quadrupolar coupling constants. For the first time, five distinct aluminum environments were resolved and individually characterized by their chemical shift and quadrupolar coupling constant values (Figure 5). DFT calculations were performed on both a cage model and the crystallographically characterized 26,9 sheet structure to assign the observed sites. The dominant signal, accounting for approximately 40% of aluminum centers, corresponds to the [AlO_3_Me] environments that constitute the backbone of the MAO framework and are common to both structural types. Two further signals in the 120–165 ppm region were assigned to bismethyl aluminum environments in which a TMA‐derived AlMe_2_ group is stabilized by a bridging methyl from a neighboring aluminum center. Critically, the sites exhibiting the highest chemical shift (∼162 ppm) and largest quadrupolar coupling constant (∼27 MHz) were identified as Lewis acidic reactive centers, on the basis that their intensity decreased markedly upon treatment with THF or addition of Cp_2_ZrMe_2_. This is consistent with earlier EPR spin probe studies using TEMPO as a Lewis base reporter, which had already identified at least two distinct types of Lewis acidic sites in MAO differing in strength and coordination environment [35, 36, 37]. It should be noted, however, that the NMR study provides no direct evidence for either the Me_2_Al^+^ abstraction or methide abstraction mechanisms operative in catalyst activation: the ionic products expected from those pathways were not detected, and the observed spectroscopic changes reflect Lewis base coordination to specific aluminum sites rather than the ionization event itself. Both the cage and sheet DFT models reproduced the observed NMR parameters with comparable accuracy, a finding the authors attributed to the fact that ^27^Al NMR probes local coordination topology, which is similar in the two structural types at sites away from the sheet edges. The power of the study therefore lies in providing the first direct, site‐resolved experimental picture of MAO's aluminum environments and their differential reactivity toward Lewis bases, an achievement made possible by the combination of ultrahigh field instrumentation and quantitative DFT prediction [38].

### 3.2 Computational IR Spectroscopy

No study has reported a computed IR spectrum of MAO as such, and the reasons are instructive. MAO in solution is a polydisperse mixture of species in dynamic equilibrium, and its experimental IR spectrum is a superposition of contributions from all components present. Assigning features in such a spectrum to specific structural models requires population‐weighted ensemble averaging over a realistic structural distribution, a computation that has not been attempted for IR, in contrast to the Boltzmann‐weighted ^27^Al NMR simulations discussed above. Without such averaging, comparison of a single model compound's computed spectrum to experiment is unlikely to yield unambiguous structural information, and the risk of confirmation bias is substantial.

Where DFT has proven genuinely useful in the IR context is not in characterizing MAO itself but in assigning bands arising from the metallocene component of MAO/metallocene mixtures. Eilertsen, Støvneng, Ystenes, and Rytter exploited the fact that the Cp ring out‐of‐plane C—H deformation mode near 800 cm^–1^ is sensitive to the bonding environment around zirconium and shifts in a systematic way with electron density on the ring and Zr–Cp distance. DFT‐calculated harmonic frequencies for a series of zirconocene species, including the neutral precatalyst, the binuclear cation, µ‐Me bridged intermediates, and the bare metallocenium, correlated well with experimentally observed band positions [39]. This correlation allowed the identification of new species formed at low Al/Zr ratios in both MAO‐ and boron‐activated systems. The study also provided DFT‐based guidance on the preferred connectivity in MAO/Cp_2_ZrCl_2_ mixtures, suggesting that Al—Cl—Al and Zr—Me—Al bridging is preferred over Al—Me—Al and Zr—Cl—Al, though the authors noted that kinetic factors might complicate this thermodynamic preference. The broader conclusion is that IR spectroscopy, while powerful for tracking the metallocene component through activation, is a blunt tool for structural assignment of MAO without authentic reference standards or ensemble‐averaged simulations.

### 3.3 Computational UV–Vis Spectroscopy

UV–vis spectroscopy occupies a distinctive niche in the characterization of MAO‐activated catalyst systems: unlike ^27^Al NMR, it is blind to the large excess of MAO present under polymerization conditions, making it selectively sensitive to the metallocene‐containing species. The relevant observable is the ligand‐to‐metal charge transfer (LMCT) transition of the zirconocene, which shifts systematically as the electronic environment around zirconium changes during activation. Computational simulation of these transitions by time‐dependent DFT has provided both interpretive support for experimental data and a sober assessment of what UV–vis can and cannot resolve in these complex mixtures.

The interpretation of UV–vis spectra in the metallocene/MAO context rests on a substantial body of experimental work that preceded computational studies. Deffieux, Cramail, and coworkers established the two‐step spectroscopic signature of metallocene activation by MAO: an initial hypsochromic shift accompanying monomethylation of the dichloride precursor, followed by a bathochromic shift upon cationization through abstraction of the second chloride [40]. The resulting species absorb light at wavelengths that are characteristic of the contact ion‐pair. Comparative studies using TMA‐depleted MAO clarified the distinct roles of TMA and MAO in these steps and demonstrated that the cationization products formed with TMA‐free activators are catalytically active at markedly lower Al/Zr ratios than those formed with commercial MAO. Brintzinger and coworkers extended this picture by identifying two spectroscopically distinct MeMAO anion types, differing in their coordinative strength toward the zirconocenium cation and in their response to added TMA [41]. Their work provided the first experimental evidence that the Lewis acidity of the MAO counterion is not uniform across the mixture. Against this experimental backdrop, the first TD‐DFT study of UV–vis spectra in this context was reported by Belelli, Damiani, and Castellani, who examined the Cp_2_ZrCl_2_/MAO system using simple MAO site models [42]. Their calculations showed that coordination to MAO redshifts the LMCT transition relative to the free metallocene and simultaneously reduces oscillator strengths. This finding is consistent with the experimentally observed changes in band position and intensity upon activation. Solvent effects, evaluated through continuum models, were found to influence the ion‐pair dissociation energetics but not to alter the qualitative picture of the LMCT shifts. The most computationally developed study of this type was reported by Velthoen, Boereboom, Bulo, and Weckhuysen, who combined UV–vis diffuse reflectance spectroscopy of five silica‐supported metallocene catalysts with varying MAO loadings with an extensive TD‐DFT library of metallocene structures [43]. Guided by computed excitation energies, spectral deconvolution allowed the authors to identify the dominant species at each loading and to propose a mechanism in which AlMe_2_^+^ species inherent to the MAO activator are responsible for initial metallocene activation. This process generates a TMA‐stabilized dormant species that releases the active cationic metallocene upon olefin insertion.

Collins and Linnolahti subsequently extended TD‐DFT UV–vis simulations to a broader range of substituted metallocene systems activated by the 16,6 sheet model, including the industrially relevant rac‐ethylenebis(indenyl)zirconium (EBIZr) family [44, 45]. Several important conclusions emerged. The longest‐wavelength LMCT transitions of the most stable neutral adducts formed between Cp_2_ZrMe_2_ and the 16,6 sheet are predicted to fall below the toluene solvent cutoff near 285 nm; these species would not be directly detectable under standard solution conditions. More significantly, the spectra of contact ion‐pairs were found to overlap with those of neutral precursors, so that inactive and active species cannot be reliably distinguished by UV–vis alone without authentic reference standards. This conclusion is directly analogous to that reached for IR spectroscopy. A positive finding was that strongly deactivated species, if present, should in principle be detectable by their distinctive spectral signatures [44].

A further complication emerged from the conformational sensitivity of the LMCT transitions. For the EBIZr system, a change of approximately 0.1 Å in the Zr—Me bond distance, which is well within the range spanned by conformational isomers, shifts the lowest‐energy LMCT by approximately 50 nm [45]. This shift is large enough to obscure meaningful comparison between a single computed structure and the experimentally observed broad envelope. Incorporating Boltzmann‐weighted averages over the relevant conformer populations and applying corrections for the systematic overestimation of excitation energies by the chosen functional brought computed spectra into good agreement with experiment for certain systems. The overall message is that UV–vis spectroscopy of MAO‐activated metallocenes is a sensitive probe of the zirconium coordination environment but one whose computational interpretation requires careful treatment of conformational flexibility. In the end, disentangling the contributions of multiple coexisting species in realistic catalyst mixtures remains beyond what UV–vis alone can achieve.

### 3.4 Total X‐Ray Scattering and the Pair Distribution Function (PDF)

Total X‐ray scattering, analyzed through the atomic PDF, offers a structural probe that is orthogonal to both mass spectrometry and NMR: rather than reporting on molecular composition or local electronic environments, it encodes the full distribution of interatomic distances in a sample, including correlations beyond the nearest‐neighbor shell. In the context of MAO, the approach amounts to comparing the experimental PDF against a library of simulated PDFs computed from DFT‐optimized structural models, ruling out structures whose simulated curves are inconsistent with experiment rather than identifying a unique solution.

The first application of PDF analysis to MAO was reported by Kilpatrick, O’Hare, and coworkers as part of a broader characterization of solid polymethylaluminoxane (sMAO), an insoluble form prepared by the controlled reaction of trimethylaluminum with benzoic acid followed by thermolysis. They generated simulated PDF curves for 36 DFT‐optimized organoaluminum structures and compared them to experimental data. Small molecule models lacked the structural dimensions to reproduce the experimental features, and the data were found to be most consistent with larger cage and nanotubular structures with *n* > 10 aluminum centers. The resolution of the data at that point was insufficient to definitively distinguish between these two motif types, but the study established the practical viability of the PDF approach for MAO and set a lower bound on the relevant structural scale [46].

A more comprehensive study was reported by Wada and Taniike, who applied synchrotron X‐ray total scattering at SPring‐8 to four distinct MAO samples. The first sample was prepared by hydrolysis, the second by thermal decomposition with benzoic acid, the third was a solid powder, and the fourth sample was a modified MAO (MMAO), alongside a dried variant. A striking first finding was that all samples produced essentially identical atomic PDF curves, with pronounced peaks at *r* ≈ 1.8 Å (Al—O and Al—C pairs), 3.2 Å (Al—Al and O—O pairs across four‐ and six‐membered rings), and weaker features at longer distances, regardless of preparation method or physical form. The inner molecular structure is therefore robust across synthetic routes, while differences between samples were confined to the small‐angle scattering region, reflecting variation in higher‐order aggregation state rather than in primary molecular structure. A library of 172 DFT‐optimized models spanning small molecules, tubes, cages, and sheets across a wide range of molecular weights was then used for systematic fitting. The conclusions were unambiguous on several counts: small molecular models and nanotubular structures failed to reproduce the experimental data regardless of their molecular weight, ruling out both classes as major components. Cage and sheet models performed comparably better, but the sheet motif showed a distinctive behavior: above a molecular weight threshold of approximately 1100 g mol^–1^, the fitted residual became independent of molecular weight for sheet structures. This molecular weight independence is a direct consequence of larger sheets growing by extending the two‐dimensional framework without fundamentally altering local topology. This behavior is consistent with a polydisperse mixture of different‐sized sheets, which would collectively produce a PDF curve that does not shift significantly with the size distribution. A polydisperse cage mixture, on the other hand, would show more pronounced size‐dependence. Sheet models containing fewer four‐membered rings provided the best reproduction of the experimental PDF curve. Furthermore, a peak observed in the small‐angle scattering region at *Q* ≈ 0.7 Å^–1^ was reproduced by stacked sheet models but not by single sheets, directly connecting the PDF evidence to the stacking motif seen in the crystallographically characterized 26,9 structure. Notably, this small‐angle feature was absent in MMAO. The isobutyl groups in MMAO confer solubility in aliphatic hydrocarbons directly through their steric and electronic influence on the aluminum framework, while their bulk simultaneously prevents sheet stacking, accounting for MMAO's enhanced stability toward gelation relative to standard MAO. Notwithstanding these positive findings, no single molecular model fully reproduced all features of the experimental curve, pointing to the coexistence of multiple structural components, a conclusion entirely in keeping with the ESI‐MS picture of MAO as a broad distribution of species [47].

Scattering methods had earlier been applied to MAO at lower resolution. Stellbrink and coworkers used small‐angle neutron scattering, complemented by static and dynamic light scattering, to characterize polymeric MAO in toluene, identifying distinct scattering regimes and providing some of the first quantitative estimates of MAO aggregate dimensions in solution [48]. These early scattering studies anticipated the aggregation behavior later resolved in atomic detail by the PDF approach.

## 4 Synthesis‐Based Approaches: TMA Hydrolysis Studies

A conceptually different approach to predicting MAO structure emerged in the mid‐2000s, shifting the focus from evaluating relative stabilities of hypothetical structures to modeling the actual formation of MAO through sequential hydrolysis of TMA. Rather than asking which structures are most stable, these studies asked which structures are most likely to form. Notably, all three groups that pioneered this approach chose MP2 over DFT as their computational method. They recognized the well‐documented failure of common density functionals such as B3LYP to reproduce the dimerization energy of TMA, the very first step in MAO chemistry [49, 50, 51]. This methodological choice proved prescient, as the limitations of DFT for aluminoxane systems extend well beyond TMA dimerization.

**SCHEME 1:**
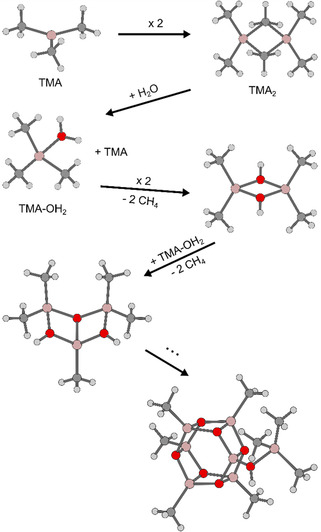
TMA hydrolysis pathways proposed by Hall and coworkers, showing the sequential condensation steps from TMA monomer through the Sinn dimer and trimer to larger oligomers. Aluminum atoms are shown in pink, oxygen in red, carbon in dark gray, and hydrogen in light gray.

The first synthesis‐based computational study was reported by Hall and coworkers in 2006, who used ab initio molecular dynamics at the MP2 level to simulate elementary steps in TMA hydrolysis (Scheme 1) [49]. Hydrolysis of the TMA dimer produces a TMA–OH_2_ complex, which undergoes intramolecular methane elimination to yield AlMe_2_OH, the bifunctional monomer for MAO growth. Coupling of these monomers with concomitant methane elimination proceeds as a step polymerization, generating dimers with four‐membered Al_2_O_2_ rings and trimers with fused ladder‐like structures. This process eventually generates a hexameric (AlOMe)_6_ cage analogous to Barron's crystallographically characterized *tert*‐butylaluminoxane [16]. A key finding emerged upon examining growth beyond this hexameric cage: insertion of additional monomers by opening strained square–square Al—O edges was thermodynamically preferred over intramolecular cage closure, yielding extended rod‐like structures through bi‐ and tri‐directional growth channels. This suggested that closed cage structures, while representing the natural products of *tert*‐butylaluminoxane chemistry, are not the favored outcome of MAO formation. This finding was an important early indication that the *tert*‐butyl analogy may be misleading.

**SCHEME 2:**
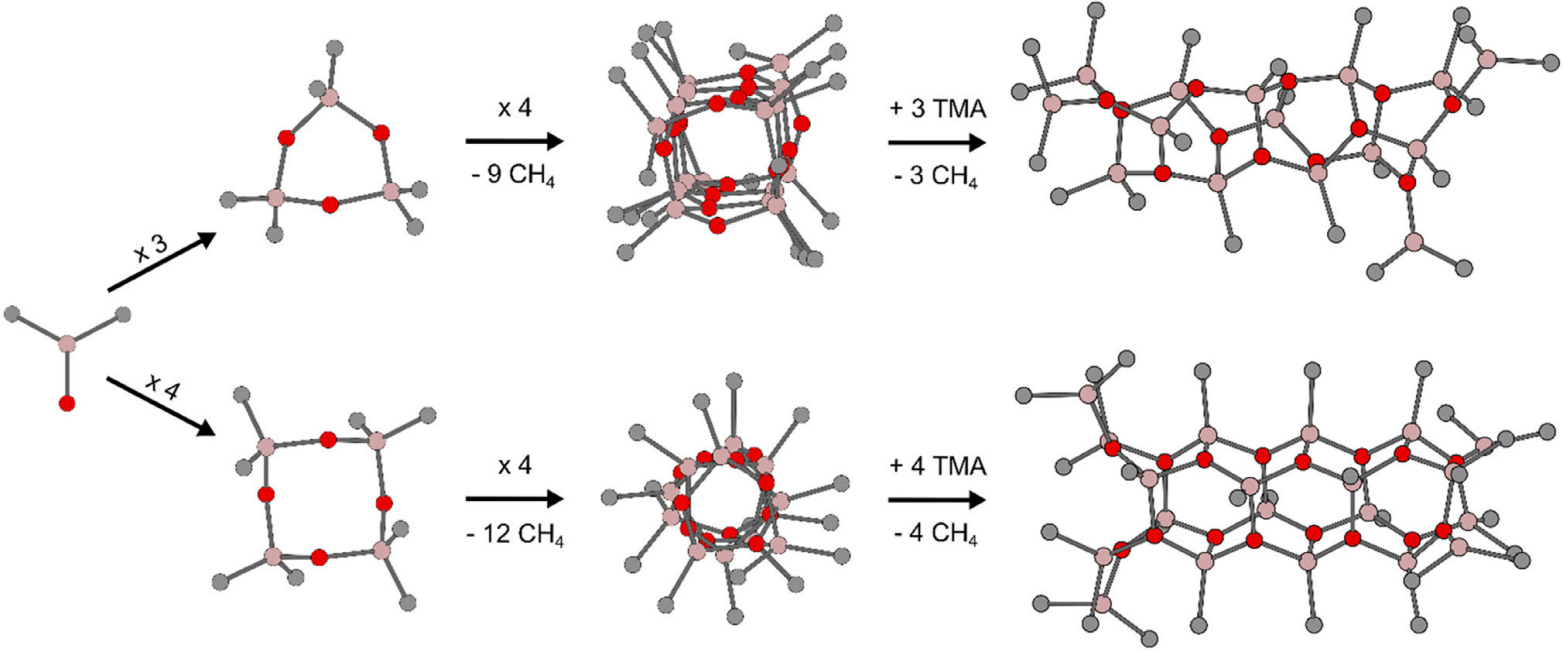
Nanotubular MAO formation pathway proposed by Linnolahti, Severn, and Pakkanen showing the coupling of cyclic trimers to form chiral (2,1) nanotubes (top) and cyclic tetramers to form armchair (2,2) nanotubes (bottom), with concomitant methane elimination. Aluminum atoms are shown in pink, oxygen in red, and carbon in gray. Hydrogen atoms are omitted for clarity.

In 2008, Linnolahti, Severn, and Pakkanen took the same AlMe_2_OH monomer as the starting point but followed a different growth logic (Scheme 2) [50]. Rather than sequential monomer addition, they considered the coupling of cyclic oligomeric rings – trimers to form chiral (2,1) nanotubes and tetramers to form armchair (2,2) nanotubes – with concomitant methane elimination. The coupling reactions are highly exothermic, and the length of the resulting tubes is determined by competition between further reaction with AlMe_2_OH and termination by TMA. The stabilities of the resulting nanotubular MAO products were compared with the truncated‐octahedral (AlOMe)_12_ cage after extracting the energy contribution of associated TMA, allowing a direct comparison of the (AlOMe)_*n*_ cores. The armchair Al_16_O_12_Me_24_ hexadecamer emerged as the thermodynamic sink, more stable than the (AlOMe)_12_ cage at room temperature. The stability advantage of the nanotube diminished with increasing temperature due to dissociation of associated TMA. This finding led to a predicted crossover around 370 K, in agreement with the experimentally observed drop in polymerization activity upon heating MAO above this temperature [52]. The paper also explicitly benchmarked B3LYP against MP2 and CCSD(T) for TMA dimerization, demonstrating a discrepancy of approximately 45 kJ mol^–1^. This dramatic failure is attributable to the lack of dispersion corrections in the density functionals available at the time, which are essential for describing the three‐center two‐electron bonds in the TMA dimer.

In 2011, Glaser and Sun provided a detailed thermochemical analysis of the very earliest steps in the hydrolysis pathway [51]. They studied methane elimination from AlMe_2_OH itself, from its dimeric aggregate, and from its TMA aggregate, all at the MP2 level. Their results established that dimerization of AlMe_2_OH is irreversible and strongly favored over mixed aggregation with TMA. Further, methane elimination from the dimer is clearly exergonic, particularly when the products are stabilized by Lewis donor coordination. The open dialuminoxane isomer was identified as a key intermediate, and a mechanism for further oligomerization through homologation of these acyclic species with AlMe_2_OH was proposed. These findings provided support for Sinn's earlier proposal regarding the formation of oligoaluminoxanes [22] and offered a rigorous thermochemical foundation for the initial stages of the process.

Together, these three studies established that the formation mechanism of MAO naturally produces extended, open structures rather than the closed polyhedral cages that had dominated structural thinking based on the *tert*‐butylaluminoxane analogy. However, each study explored only selected reaction pathways, and a comprehensive, systematic investigation of all possible hydrolysis products and their relative stabilities remained to be undertaken.

The first systematic computational screening of TMA hydrolysis products was reported in 2013, mapping out the complete structural landscape at small oligomer sizes [53]. The product space was defined using the general formula (AlOMe)_*n*_(AlMe_3_)_*m*_, where *n* counts the number of hydrolysis equivalents and *m* the number of associated TMA units. All thermodynamically accessible products of TMA hydrolysis can be located on a two‐dimensional (*n*,*m*) grid, and the approach amounts to finding the most stable structure at each grid point rather than selecting candidates based on chemical intuition. To enable direct stability comparison across all compositions, the Gibbs free energy of the balanced hydrolysis reaction 0.5(*n* + *m*)Al_2_Me_6_ + *n*H_2_O → (AlOMe)_*n*_(AlMe_3_)_*m*_ + 2*n*CH_4_ was evaluated and normalized per hydrolysis equivalent (Table 1), yielding Δ*G*/*n* as a universal thermodynamic measure. With TMA and water as common references, this normalization allows structures of different size and composition to be ranked on a single scale and became the standard stability metric in all subsequent work from our group. The method was applied systematically up to *n* = 4 and *m* = 4 at the MP2/def‐TZVP level, yielding a comprehensive library of optimized structures.

**TABLE 1:** Gibbs free energies (Δ*_r_G*, kJ mol^–1^) and relative Gibbs free energies (Δ(Δ*_r_G*), kJ mol^–1^) of the balanced hydrolysis reaction 0.5(*n *+ *m*)Al_2_Me_6_ + *n*H_2_O → (AlOMe)_*n*_(AlMe_3_)_*m*_ + 2*n*CH_4_ for MAO structures with *n* = 1–4, calculated at MP2/def‐TZVP. Values of Δ(Δ*_r_G*) are given relative to *n* = 4, *m* = 4.

*n*	*m*	Formula	Δ*_r_* *G*	Δ(Δ*_r_* *G*)
1	0	AlMeO	−20.9	380.6
1	1	Al_2_Me_4_O	−297.5	104.0
1	2	Al_3_Me_7_O	−373.1	28.3
1	3	Al_4_Me_10_O	−381.7	19.7
1	4	Al_5_Me_13_O	−363.2	38.2
2	0	Al_2_Me_2_O_2_	−439.8	181.6
2	1	Al_3_Me_5_O_2_	−587.4	107.8
2	2	Al_4_Me_8_O_2_	−756.5	23.2
2	3	Al_5_Me_11_O_2_	−769.8	16.6
2	4	Al_6_Me_14_O_2_	−776.6	13.2
2	5	Al_7_Me_17_O_2_	−750.1	26.4
3	0	Al_3_Me_3_O_3_	−863.7	113.6
3	1	Al_4_Me_6_O_3_	−984.4	73.3
3	2	Al_5_Me_9_O_3_	−1100.1	34.8
3	3	Al_6_Me_12_O_3_	−1179.7	8.2
3	4	Al_7_Me_15_O_3_	−1177.6	8.9
4	0	Al_4_Me_4_O_4_	−1305.8	75.0
4	1	Al_5_Me_7_O_4_	−1391.6	53.6
4	2	Al_6_Me_10_O_4_	−1491.4	28.6
4	3	Al_7_Me_13_O_4_	−1592.3	3.4
4	4	Al_8_Me_16_O_4_	−1605.8	0.0
4	5	Al_9_Me_19_O_4_	−1562.4	10.9

*Note:* This normalization per hydrolysis equivalent provides a universal thermodynamic stability measure allowing direct comparison across compositions and sizes. Reproduced with permission [53]. Copyright 2013, WILEY‐VCH Verlag GmbH & Co. KGaA, Weinheim.

The structural evolution across the (*n*,*m*) grid reveals how associated TMA progressively transforms the aluminoxane framework. At *m* = 0, the familiar (AlOMe)_*n*_ cores are recovered: the four‐membered Al_2_O_2_ ring at *n* = 2, the hexagonal Al_3_O_3_ ring at *n* = 3, and the cubic (AlOMe)_4_ cage at *n* = 4. Association with one equivalent of TMA opens strained Al—O bonds and introduces Al—Me—Al bridging methyl groups, consistent with the structural motifs proposed by Ystenes and Rytter [24, 54]. As *m* increases further, the structures evolve from compact cages toward progressively more open topologies: rings and chains at *n* = 2 and 3, and at *n* = 4 from the cuboidal cage through intermediate geometries to a sheet‐like structure at *m* = 3, the only sheet motif encountered in this size domain. The Δ*G*/*n* values decrease monotonically with increasing *n*, confirming the thermodynamic driving force toward larger oligomers. The optimal *m* at a given *n* reflects the balance between the energetic gain of relieving ring strain through TMA association and the entropic cost of incorporating additional molecules.

Some of these small hydrolysis products have been characterized experimentally. Kacprzak and Serwatowski showed by multinuclear NMR and cryoscopic molecular weight determination that tetramethyldialuminoxane, the 1,1 chain, exists as a cyclic trimer (Me_4_Al_2_O)_3_ in solution [55]. This trimer has an Al_3_O_3_ ring core and Al—Me—Al bridging methyl groups, corresponding to the 3,3 ring on the grid. However, tetramerization to the 4,4 ring, predicted to be thermodynamically more stable, cannot be ruled out by the available data. When Lewis base donors are present, the oligomerization is arrested at an earlier stage. Lewiński and coworkers isolated the pyridine‐stabilized dimer [AlMe_2_(pyr)(μ_3_‐O)AlMe_2_]_2_ by controlled hydrolysis in the presence of pyridine [56], while Berger and Anwander crystallized the THF‐stabilized analog [(Me_2_Al)_2_O(thf)]_2_ from low‐temperature hydrolysis in THF [57]. Both structures correspond to the 2,2 composition. These experimental observations provide direct validation of the smallest structures on the (*n*,*m*) grid and confirm that the (AlOMe)_*n*_ core with associated TMA framework captures the essential chemistry of early MAO formation.

Extending the systematic study to *n* = 5–8, the conformational space was found to expand rapidly beyond the reach of exhaustive enumeration [58]. While the pentamers (*n* = 5) were still tractable by systematically considering all elementary reactions between every atom and bond, the larger oligomers (*n* = 6–8) required a different strategy: new structures were built sequentially from the lowest‐energy smaller ones. Two distinct sheet architectures emerged in this size domain. The first, already hinted at by the 4,3 structure reported in the preceding study [53], features five‐coordinate aluminum and becomes the preferred motif at smaller TMA loadings. The second type, favored at higher TMA association, consists exclusively of four‐coordinate aluminum and predominantly three‐coordinate oxygen, with edge sites where the associated TMA introduces four‐coordinate oxygen environments. These four‐coordinate sheets share a structural principle with Atwood's experimentally characterized [Al_7_O_6_Me_16_]^−^ anion [15]: both are built from fused six‐membered Al_3_O_3_ rings with four‐coordinate aluminum and three‐coordinate oxygen. The main difference between these structures is size: the Atwood anion comprises three fused rings, while the thermodynamically preferred 8,5 sheet contains four. Thermodynamically, the four‐coordinate sheets are slightly preferred over the five‐coordinate sheets at *n* = 8. However, both remain more stable than cage structures of comparable size, indicating that the sheet‐to‐cage crossover lies beyond this size regime.

Extending the sequential growth approach to the experimental size domain, the structural evolution continued from sheets toward cage‐like structures, with the sheet‐to‐cage transition predicted to occur at *n* = 13 [59]. Beyond this point, cages of increasing size became progressively more stable, with the thermodynamic minimum found at 16,6 with an elongated, tubular structure. From the Boltzmann distribution of relative stabilities, 16,6 emerged as the dominant species, in equilibrium with 16,5 and lesser amounts of 17,6 and 18,6. The resulting average molecular weight is 1163 g mol^–1^ with an empirical composition of (Me_1.48_AlO_0.76_)_*n*_, in reasonable agreement with experimental estimates [21]. This prediction found support in ESI‐MS studies by the University of Victoria group, which identified the anion [(MeAlO)_16_(AlMe_3_)_6_Me]^−^, hereafter [16,6]^−^, as the overwhelmingly dominant species in fresh MAO solutions, with [17,6]^−^ and [18,6]^−^ as minor components [9]. An earlier report identifying [23,7]^−^ as dominant [7] was subsequently shown to reflect analysis of aged material, with the ion distribution shifting toward higher molecular weight upon prolonged storage [8]. The apparent agreement between computational prediction and experimental observation lent credibility to the 16,6 cage as a working model for MAO, and it was subsequently adopted in further studies [60, 61, 62, 63]. Notably, Zaccaria and coworkers used it as the basis for a systematic DFT study of Lewis acidic site reactivity, identifying three distinct site types with markedly different tendencies to release AlMe_2_^+^ [64].

**FIGURE 6:**
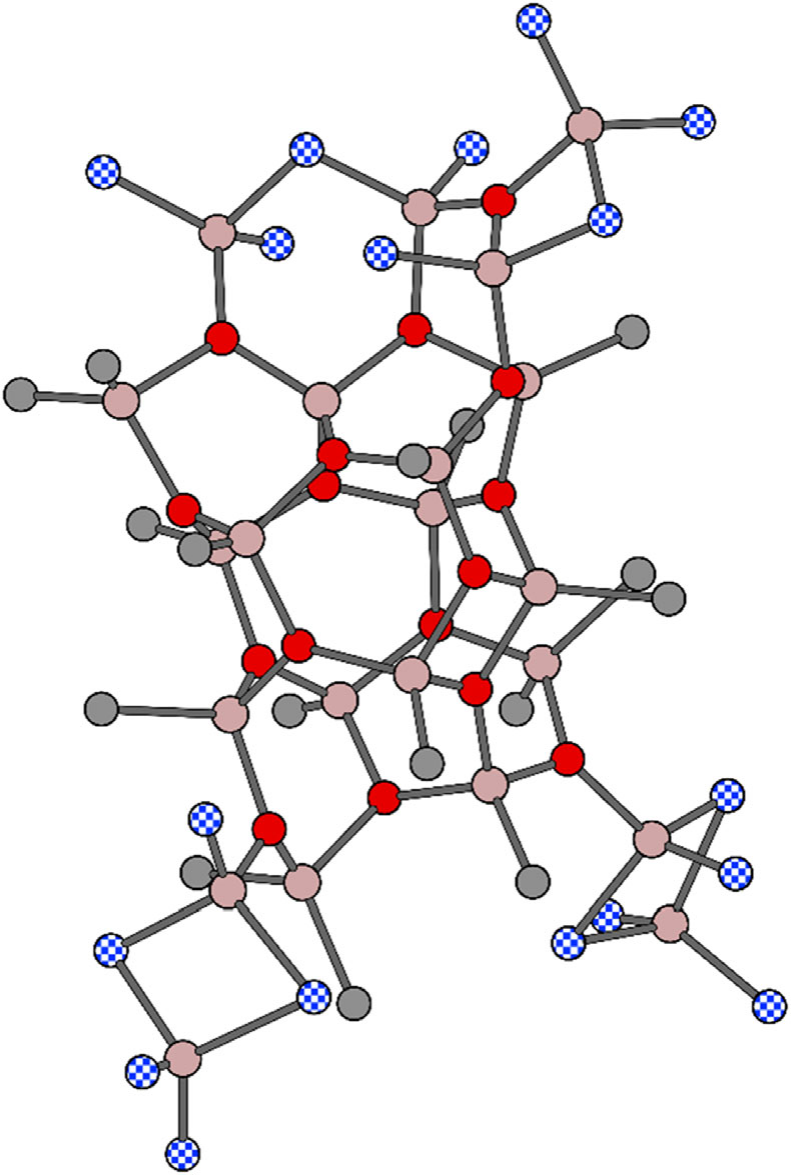
Structure of the most stable 16,6 cage model located in the hydrolysis studies, showing the 18 labile methyl sites (highlighted in checkered blue) available for alkyl exchange. The experimentally observed extent of methyl‐for‐ethyl exchange exceeding 20–22 substitutions is inconsistent with this architecture. Aluminum atoms are shown in pink, oxygen in red, and carbon in gray. Hydrogen atoms are omitted for clarity.

The credibility of the 16,6 cage model was, however, soon undermined by experiment. An ESI‐MS study of MAO reactivity toward trialkylaluminum reagents (R_3_Al, R = Et, iBu, nOct) revealed that the extent and selectivity of alkyl exchange were inconsistent with a cage or nanotube architecture [62]. In the case of Et_3_Al, methyl‐for‐ethyl exchanges on the [16,6]^−^ anion extended to at least 20–22 substitutions, far exceeding the 18 labile methyl positions available in the 16,6 cage (Figure 6). This finding was clearly inconsistent with a cage structural assignment. Instead, the results pointed toward structures with an unusually high proportion of edge sites per molecule, leading the authors to conclude that “the MAO activator(s) are likely to have unusual structures that depart significantly from the cage‐like motifs or even nanotubes that have been considered so far.” This experimental finding forced a fundamental reexamination of the computational approach that had led to the cage prediction, which is the subject of the following section.

## 5 Methodological Considerations

Extending the systematic study of MAO oligomers to the experimentally relevant size domain required a transition away from MP2, as the computational cost of correlated ab initio methods became prohibitive for systems with 20 or more aluminum centers. The choice of a suitable DFT functional for this task was not straightforward: the early and comprehensive benchmarking study by Willis and Jensen demonstrated that B3LYP underestimates the TMA dimerization enthalpy by more than 54 kJ mol^–1^ relative to the experimental value of 85.3 ± 1.4 kJ mol^–1^, while MP2 reproduces it to within approximately 8 kJ mol^–1^ [65]. This fundamental difficulty with bridging Al—C environments had long made DFT a questionable choice for MAO energetics.

The situation improved considerably with the development of the Minnesota family of functionals [66, 67, 68, 69]. A comprehensive benchmarking study of DFT methods for dimerization and complexation energies in systems relevant to Ziegler–Natta catalysis and related aluminum chemistry by Ehm and coworkers identified M06‐2X as the top‐performing functional [70]. It achieved a mean absolute deviation of only 2.5 kJ mol^–1^ against CCSD(T) reference values for a test set of 26 bridged aluminum and transition metal systems, including the TMA dimerization. Paired with a triple‐ζ basis set (TZVP), M06‐2X offered a cost‐effective route to reliable energetics for the Al—C bridging environments characteristic of MAO edge sites, and it became the standard functional for computational MAO studies.

Later studies, however, uncovered that M06‐2X carries a systematic error for a structural environment that is prevalent in sheets but largely absent in cages: the four‐coordinate Al—O bond. As demonstrated in recent work comparing M06‐2X and MP2 predictions for small MAO oligomers, M06‐2X artificially stabilizes structures containing four‐coordinate oxygen [12]. This failure lies outside the benchmark for TMA dimerization, which involves an Al—C bridged environment, and thus went undetected for an extended period. The performance of M06‐2X on the benchmark that had motivated its adoption gave no warning of its inadequacy for the structural motif that turned out to be thermodynamically decisive.

A more fundamental source of error lies in the treatment of entropy. Sheets and cages are not merely geometric isomers: they differ profoundly in the nature of their low‐frequency collective vibrations. The more open and flexible sheet structures possess more low‐frequency modes than the rigid, closed cage structures, and consequently sheets have higher vibrational entropies. This difference was not recognized in the 2017 work [59], where path selection through the large oligomer space was conducted based on electronic energy rather than Gibbs free energy, systematically biasing the search toward cage‐like pathways. The magnitude of this error became quantitatively clear in subsequent work directly comparing isomeric sheets and cages at the 16,6 and 16,7 compositions. For 16,6, the cage is lower in electronic energy than the sheet by approximately 9–13 kJ mol^–1^ depending on the functional, consistent with the energy‐based path selection favoring cages. Once vibrational entropy is included, however, the sheet becomes lower in Gibbs free energy by about 40 kJ mol^–1^, representing a total reversal of about 50 kJ mol^–1^ relative to the electronic energy [71]. The electronic energy error from M06‐2X contributes only a minor fraction of this reversal; the dominant driving force is vibrational entropy, which in the experimentally relevant size domain far outweighs differences in electronic energy or zero‐point correction.

Accurate computation of this entropy contribution is itself a significant challenge. The normal modes that contribute most to vibrational entropy are the low‐frequency ones, yet these are precisely the modes that cannot be computed reliably within the harmonic oscillator approximation. Even among conformers of the same system, small numerical differences in the optimization procedure can shift the lowest mode substantially, producing large and spurious changes in the computed entropy. For MAO structures in the experimental size domain, this numerical noise in the *T*Δ*S* term is sufficient to obscure genuine thermodynamic preferences between closely competing structures. A standard remedy is Truhlar's quasi‐harmonic approximation, which eliminates this noise by raising all modes below a threshold frequency (typically 100 cm^–1^) to exactly that value before computing entropy [72]. However, this noise elimination comes with a price: the raised frequencies carry no physical meaning for the modes they replace. This misrepresentation leads to unsystematic errors in entropy for the two types of low‐frequency vibrations relevant to MAO, torsions and out‐of‐plane bends, which exhibit distinct energy level characteristics, as demonstrated recently for small aluminum systems [73]. While the quasi‐harmonic approximation alleviates the error for torsions, it systematically underestimates the entropy of low‐frequency bending motions, which constitute the majority of MAO low‐frequency modes. Although these errors can be significant even for individual modes, this uncertainty grows substantially with system size: more than 30 normal modes fall below the 100 cm^–1^ threshold in 16,6 structures.

For the computational studies of MAO crystal structure, Luo and coworkers applied a more rigorous anharmonic correction [6]. They employed the independent mode approximation of Beste [74, 75] where fourth‐order polynomial potentials are fitted along Cartesian displacement vectors, and the partition functions are solved through the semiclassical Wigner–Kirkwood method [76, 77]. This approach results in improved contributions for low‐frequency bending vibrations over the harmonic and quasi‐harmonic models, but also increases the computational cost considerably, especially for larger systems. Furthermore, the applicability of the method for MAO torsional vibrations requires validation, as these motions cannot be described properly through Cartesian displacement vectors. What makes MAO low‐frequency vibrations particularly challenging to treat is the mixing of torsions with bends; the adequate treatment of these mixed vibrations requires further research.

A final complication to accurate entropy computation arises from the solution environment in which MAO chemistry occurs. To approximate bulk solvent effects on potential energy surfaces, polarizable continuum models have been widely used in MAO studies; however, these models primarily address the electrostatic component of solvation and leave the entropic consequences of moving from gas phase to solution unaddressed. Dissolving a molecule in a liquid restricts its translational freedom relative to the gas phase, reducing the total entropy and hence the entropic cost of association reactions. In polymerization catalysis studies, this effect has been approximated by multiplying the entire *T*Δ*S* term by a factor of 2/3 [78, 79, 80], following a suggestion by Tobisch and Ziegler [81] based on the observation that the solvation entropy of ethylene in aromatic hydrocarbon solvents is approximately 67 J mol^–1^ K^–1^, roughly two‐thirds of its calculated gas‐phase value. This correction is physically motivated for systems where the reaction entropy is dominated by the translational contribution of a small molecule, such as ethylene coordination to a metal center. It is not applicable, however, to MAO in the experimental size domain. For large oligomers, vibrational entropy is the dominant contributor to total entropy and is relatively insensitive to phase [72]: the principal change on going from gas phase to solution is the suppression of translational entropy. Applying a uniform 2/3 factor to the entire *T*Δ*S* term therefore suppresses the vibrational contribution, which should be largely unchanged, while providing at best a rough correction to the translational term where the genuine phase effect resides. Furthermore, the 2/3 factor is not universal: factors of 1/2 have been proposed in other contexts, and a systematic comparison of entropy calculation methods by Talarico and coworkers found the approach neither general nor reliably accurate [82].

In subsequent work from our group [83], the solvation correction was therefore targeted specifically at translational entropy using the free volume method of Whitesides and coworkers [84], in which the Sackur–Tetrode expression is corrected by calculating a more realistic value for volume based on the density of the solvent and the size of the solute molecule. This approach belongs to a broader family of closely related solvation entropy treatments. Among the earliest and most widely applied is the model of Martin and coworkers [85], developed essentially concurrently with the Whitesides approach, which requires only the solvent density as input and has been extensively benchmarked across diverse chemical systems. More advanced models of Fang [86] and Garza [87] determine the free volume through the space available for the solute molecule within the solvent cavity, with Garza's model additionally including corrections for rotation and cavity formation. Garza's model was applied by Luo and coworkers in their computational studies of the MAO crystal structure [6]. Conquest and coworkers examined Garza's model for molecules up to 53 atoms, finding that different approaches to determining molecular volume and cavity shape influence the thermodynamic outcome, particularly for larger molecules [88]. Overall, choosing the appropriate solvation entropy treatment for MAO is not straightforward, given the lack of direct experimental comparison data and the fact that MAO oligomers in the experimentally relevant size domain are substantially larger than the systems considered in most benchmarks. Owing to the uncertainties in both low‐frequency vibrational contributions and solvation entropy, computing reliable solution‐phase Gibbs energies for systems of this size and flexibility remains an open methodological problem.

Underlying all of the specific methodological issues discussed above is a more general limitation of the static computational approach itself. The methods considered here characterize individual optimized structures, or small ensembles of them, at or near 0 K. MAO, by contrast, is a highly fluxional material at the temperatures of synthesis and catalysis, comprising an interconverting population of species in dynamic equilibrium, each surrounded by a fluctuating solvent environment. The energy differences between competing structures and pathways are frequently small, and the configurational averaging over both solute conformations and solvent environments that determines the true thermodynamic populations is not captured by static calculations on frozen clusters. A faithful treatment of this fluxionality would require molecular dynamics rather than static electronic structure methods. One consequence deserves particular emphasis: the distribution of MAO species depends sensitively on how the material is synthesized and aged, and these differences arise from precisely the kind of small energetic and dynamic effects that static methods cannot reliably resolve. Computational approaches are therefore poorly suited to discriminating between the active species generated by different synthetic routes. This limitation is mitigated, though not removed, by the relatively well‐defined nature of the discrete MAO clusters that form the basis of the structural models reviewed here, and it applies in some measure to all of the calculations discussed.

## 6 Sheet Structures of MAO

The methodological shift from electronic energy to Gibbs free energy as a path selection criterion and to a targeted search focusing separately on sheet and cage motifs was directly productive. When the hydrolysis reaction network for (MeAlO)_*n*_(AlMe_3_)_*m*_ oligomers was explored using condensed‐phase corrected Gibbs free energies as the guide, and sheet pathways were followed explicitly across the range *n* = 4–18, sheets emerged as the thermodynamically preferred structural motif throughout the size domain relevant to experiment [89]. The DFT calculations suggested a structural transition with increasing oligomer size: smaller structures (roughly *n*  ≤ 9, depending on *m*) appeared to favor five‐coordinate Al sheets featuring strained four‐membered Al_2_O_2_ rings, while larger structures were predicted to adopt four‐coordinate Al sheets built from six‐membered Al_3_O_3_ rings. However, subsequent higher‐level calculations revealed this transition to be a methodological artifact. M06‐2X artificially stabilizes structures containing four‐coordinate oxygen atoms, leading to systematic overstabilization of the five‐coordinate motif for smaller oligomers where both structural types compete. When MP2 and CCSD(T) methods are applied, the four‐coordinate Al sheets are preferred throughout the size range, and five‐coordinate Al structures are not thermodynamically relevant [12]. This finding has significant implications for understanding MAO reactivity, as the two sheet types have different edge environments and reactive sites for catalyst activation. Across the entire range investigated at the appropriate level of theory, the previously located cage structures remain higher in Gibbs free energy than their four‐coordinate sheet isomers.

**FIGURE 7:**
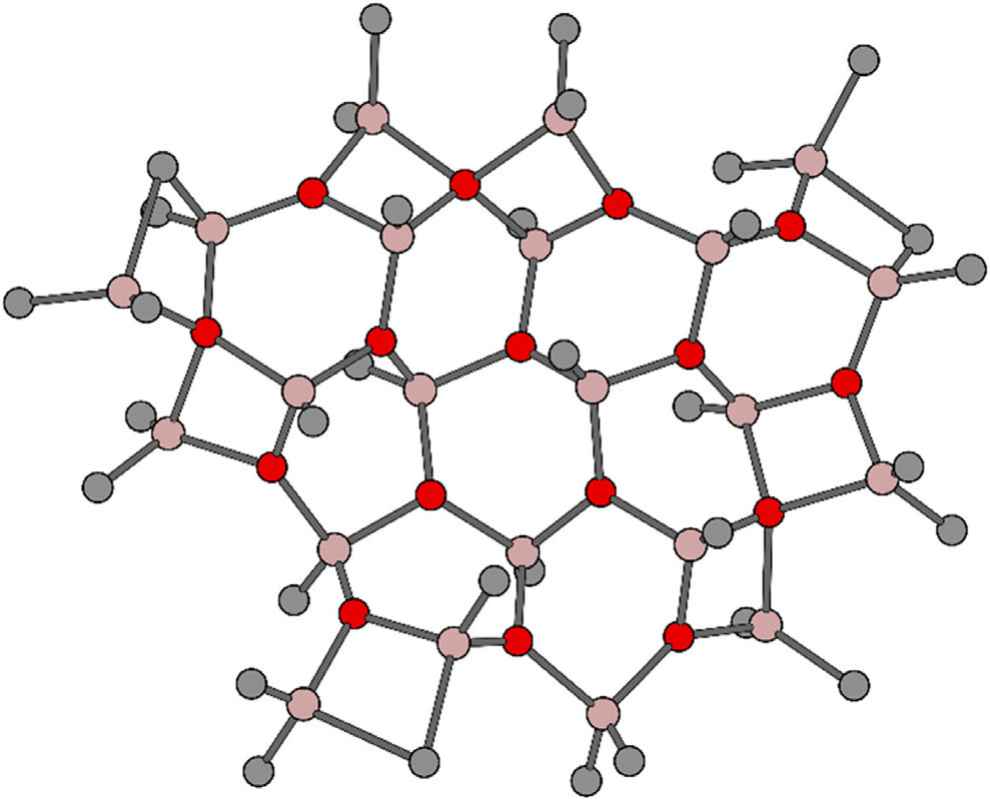
Structure of the most stable 16,6 MAO sheet located via the hydrolysis route, showing the hexagonal four‐coordinate aluminum core and the chelated Me_3_Al group at the edge forming a six‐membered ring. The 24 potentially labile edge methyl groups are consistent with the experimentally observed extent of methyl‐for‐ethyl exchange. Aluminum atoms are shown in pink, oxygen in red, and carbon in gray. Hydrogen atoms are omitted for clarity.

The thermodynamic preference of sheets over cages found a striking expression in the structure of the dominant anionic species observed in ESI‐MS experiments. The [16,6]^−^ anion that had been observed as the principal product in commercial MAO samples for years and whose abundance had never been satisfactorily explained, was shown to possess a sheet structure characterized by a hexagonal, four‐coordinate Al core with chelation of one Me_3_Al group at the edge, forming a six‐membered ring in resemblance of the bulk sheet interior (Figure 7) [90]. This new structural model was located by following the sheet‐focused hydrolysis pathway to the 16,6 composition and performing anion calculations from the resulting neutral. Comparison to the previously accepted cage anion models was unambiguous: the sheet [16,6]^−^ is lower in electronic energy by up to 66 kJ mol^–1^, lower in gas‐phase Gibbs free energy by 94 kJ mol^–1^, and lower in condensed‐phase Gibbs free energy by 86 kJ mol^–1^ relative to the most stable cage anion previously reported. As will be discussed below, this structure itself was later superseded by a still more stable [16,6]^−^ model identified through topological reasoning [91], but it represented a decisive step in establishing that the dominant activated species in fresh MAO are sheet‐based rather than cage‐based. A structural feature of the sheet anion that connected naturally to earlier experimental observations was the availability of 24 potentially labile edge methyl groups. This number is substantially larger than what the cage models could accommodate, and consistent with the broad distribution of methyl‐for‐ethyl exchanges observed experimentally, extending to at least 20–22 substitutions.

These computational findings were developed in close conjunction with real‐time ESI‐MS monitoring of MAO synthesis [11]. Following the hydrolysis of TMA in dilute fluoroarene solution from the first minute of reaction onward revealed that growth does not proceed by the stepwise, incremental addition of small aluminum‐containing units that a classical condensation mechanism would suggest. Instead, the earliest detectable species are small anions in the [7,4]^−^ to [9,4]^−^ range, which appear transiently and decay within minutes, followed by a much slower emergence of larger species ultimately dominated by [16,6]^−^. The sparse population of intermediate ions between these two size regimes points toward a growth mechanism that involves aggregation and fusion of smaller sheet fragments rather than sequential monomer addition. DFT calculations combined with kinetic simulation identified at least three distinct growth phases [92]. The first is rapid initial hydrolysis of TMA to low molecular weight linear oligomers, principally the Sinn dimer and trimer stabilized by coordination of Me_3_Al. These oligomers then assemble into small sheets, followed by a slower aging process where small sheets grow further either by addition of low molecular weight fragments along their reactive edges or by mutual fusion of larger sheets. A notable and experimentally confirmed prediction of this aggregation mechanism is the preference for even‐numbered sheets. Because two termination steps along a reactive edge are required to cap an even‐membered sheet compared to one for an odd‐membered sheet, and because fusion of two even‐numbered sheets again yields an even‐numbered product, the kinetics of growth systematically favor even values of *n*. This is precisely what is observed in ESI‐MS monitoring experiments, where odd‐numbered anions such as [15,6]^−^ and [17,6]^−^ convert progressively to their even‐numbered neighbors at the longest reaction times.

The ionization chemistry of neutral sheet precursors was subsequently analyzed in greater depth across the *n* = 1–8 range using the hierarchy of methods described above. This analysis encompassed both Me^‐^ abstraction and [Me_2_Al]^+^ abstraction pathways and incorporated free volume entropy corrections in fluorobenzene medium [12]. A central finding was that regardless of the initial ionization site or mechanism, the resulting anions consistently converge to structures featuring four‐coordinate Al and predominantly three‐coordinate O, the signature coordination environment of bulk MAO. In solution, ionization energies approach zero or become negative for reactive sheet compositions, consistent with facile ion‐pair formation under experimental conditions. Combining the free energy of hydrolysis for the neutral precursor with the solution‐phase ionization free energy reproduces the experimental pattern convincingly: it correctly identifies [4,3‐OH]^−^ and [5,3]^−^ as the weakest anions at any stage of hydrolysis, and [7,4]^−^ or [8,4]^−^ as the most intense at early reaction times depending on experimental conditions.

Between the initial computational prediction of sheet structures in 2021 and this more comprehensive ionization analysis, the sheet structural motif received decisive experimental support through the isolation and crystallographic characterization of the large 26,9 sheet by Luo et al. in 2024 [6]. They reported the first direct structural confirmation that extended two‐dimensional sheets are genuine components of MAO. This species was therefore included in the ionization analysis for comparison [12]. The result was unexpected: the 26,9 sheet is predicted to be approximately four orders of magnitude more reactive toward OMTS than the lowest energy 16,6 sheet identified at the time. Yet, ESI‐MS spectra of commercial MAO show the opposite, with [16,6]^−^ dominating and [26,8]^−^ barely detectable. No satisfactory explanation for this discrepancy could be offered at the time.

**FIGURE 8:**
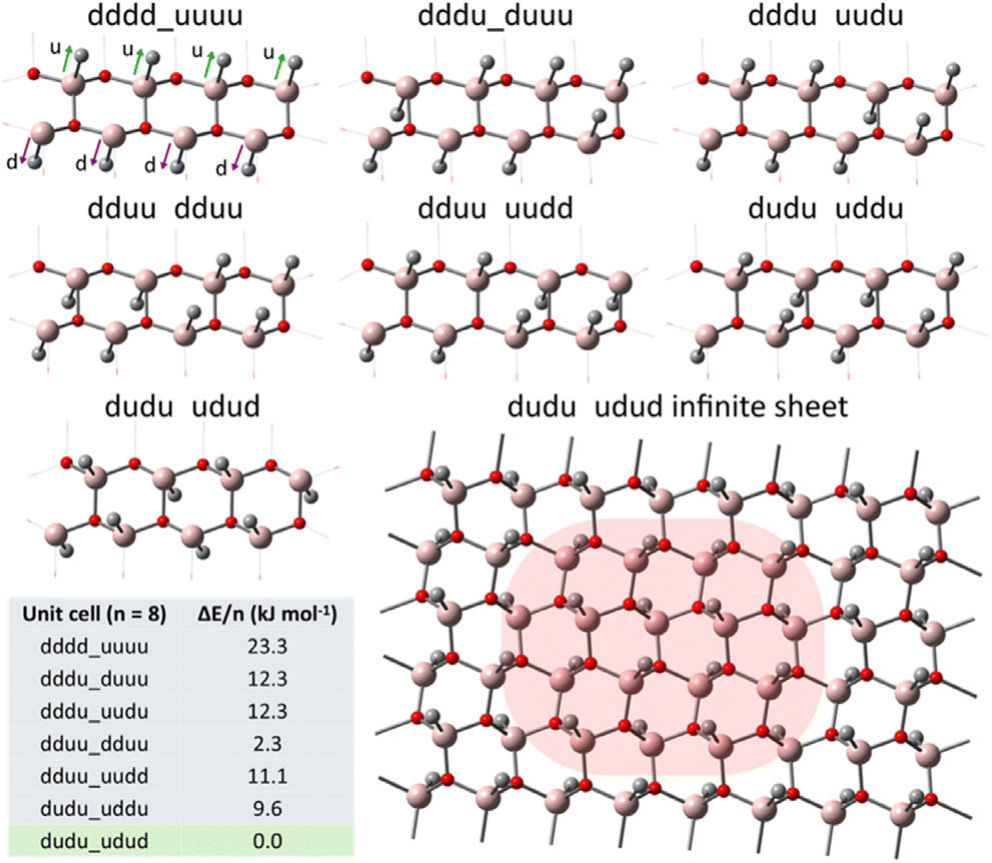
Possible methyl group arrangements in the infinite (MeAlO) sheet and their relative energies (Δ*E*/*n*, kJ mol^–1^) for an eight‐unit cell. The alternating dudu_udud arrangement is the most stable, mirroring the graphane pattern of strictly alternating up–down substituent orientations. Aluminum atoms are shown in pink, oxygen in red, and carbon in gray. Hydrogen atoms are omitted for clarity. Reproduced with permission [91]. Copyright 2026, the Royal Society of Chemistry.

The resolution came from abandoning the TMA hydrolysis pathway as the route to structural models and instead exploiting the long‐established analogy between hydrocarbons and aluminoxanes. Rooted in the isovalent electronic relationship between Al—O and C—C units, this analogy had originally been developed to rationalize the structures of aluminoxane chains, rings, and cages in terms of their hydrocarbon counterparts. The analogy extended from the allene analog of Al_2_OH_4_ through the benzene analog of Al_3_O_3_H_3_ to fullerene‐like cage structures [93, 94]. The same framework has illuminated why certain hydrogenated fullerenes are exceptionally stable through “in–out” isomerism, where alternating substituent orientations minimize steric repulsions [95]. This principle generalizes to graphane, the fully hydrogenated form of graphene, in which hydrogen atoms adopt a strictly alternating up–down arrangement relative to the plane. Recognizing that methyl groups on aluminum in a MAO sheet occupy precisely the same topological role as hydrogen atoms on carbon in graphane, it followed that the optimal arrangement of methyl groups in an infinite (MeAlO) sheet should mirror this alternating pattern. Systematic periodic calculations on an eight‐unit cell confirmed this expectation, identifying a clear energetic preference for the alternating methyl configuration (Figure 8) [91].

**FIGURE 9:**
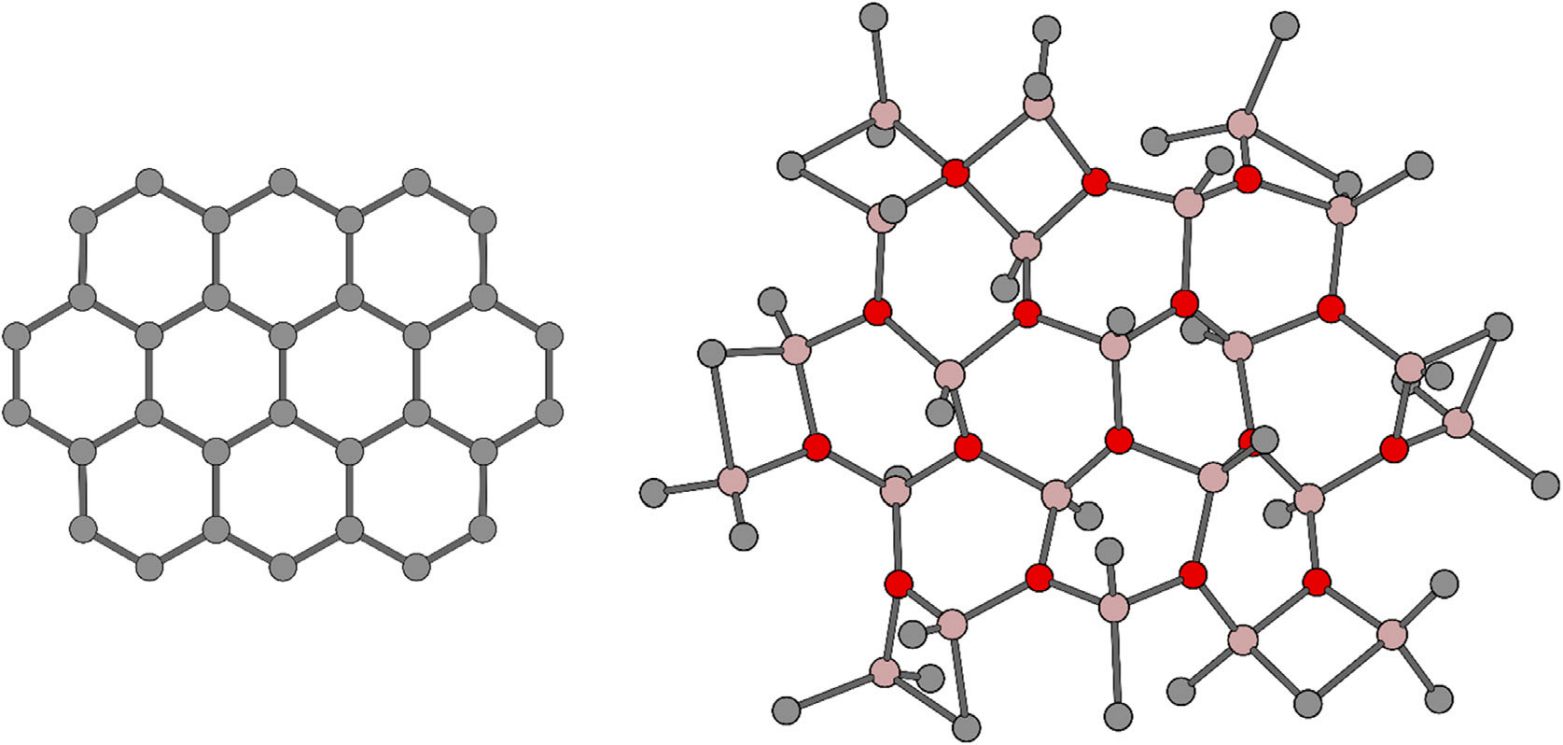
Structure of ovalene (left) and the ovalene‐analogous 16,7 MAO sheet (right), illustrating the topological correspondence between the polycyclic aromatic hydrocarbon framework and the (MeAlO) sheet. Aluminum atoms are shown in pink, oxygen in red, and carbon in gray. Hydrogen atoms are omitted for clarity.

The critical step was then to extract a finite molecular fragment from this optimized infinite sheet using an appropriate topological template. Ovalene, a polycyclic aromatic hydrocarbon consisting of 32 carbon atoms in ten fused six‐membered rings, provides the ideal framework: its aluminoxane analog yields a neutral species of composition 16,7, which can form the [16,6]^−^ anion through [Me_2_Al]^+^ abstraction (Figure 9). All symmetry‐unique ways of extracting such a fragment from the infinite sheet were explored, and the resulting structural isomers optimized. Importantly, the structural principles that govern the infinite sheet required significant adaptation at the molecular scale: the nine framework methyl groups in the finite 16,7 molecule cannot adopt a perfectly alternating arrangement. This imperfection happens because the odd count precludes the symmetric up–down balance of the infinite sheet, and the TMA edge groups introduce additional constraints absent in the periodic model. Consequently, the lowest‐energy isomer deviates from the pattern suggested by the infinite sheet, with local geometry optimized around edge interactions rather than bulk periodicity. Further configurational diversity arises at the edge sites, where the Me_2_AlO groups can coordinate either through a bridging methyl, maintaining three‐coordinate oxygen, or directly through oxygen, creating four‐coordinate oxygen, with minimal barriers between the two states. The fully three‐coordinate oxygen configuration is preferred [91].

**FIGURE 10:**
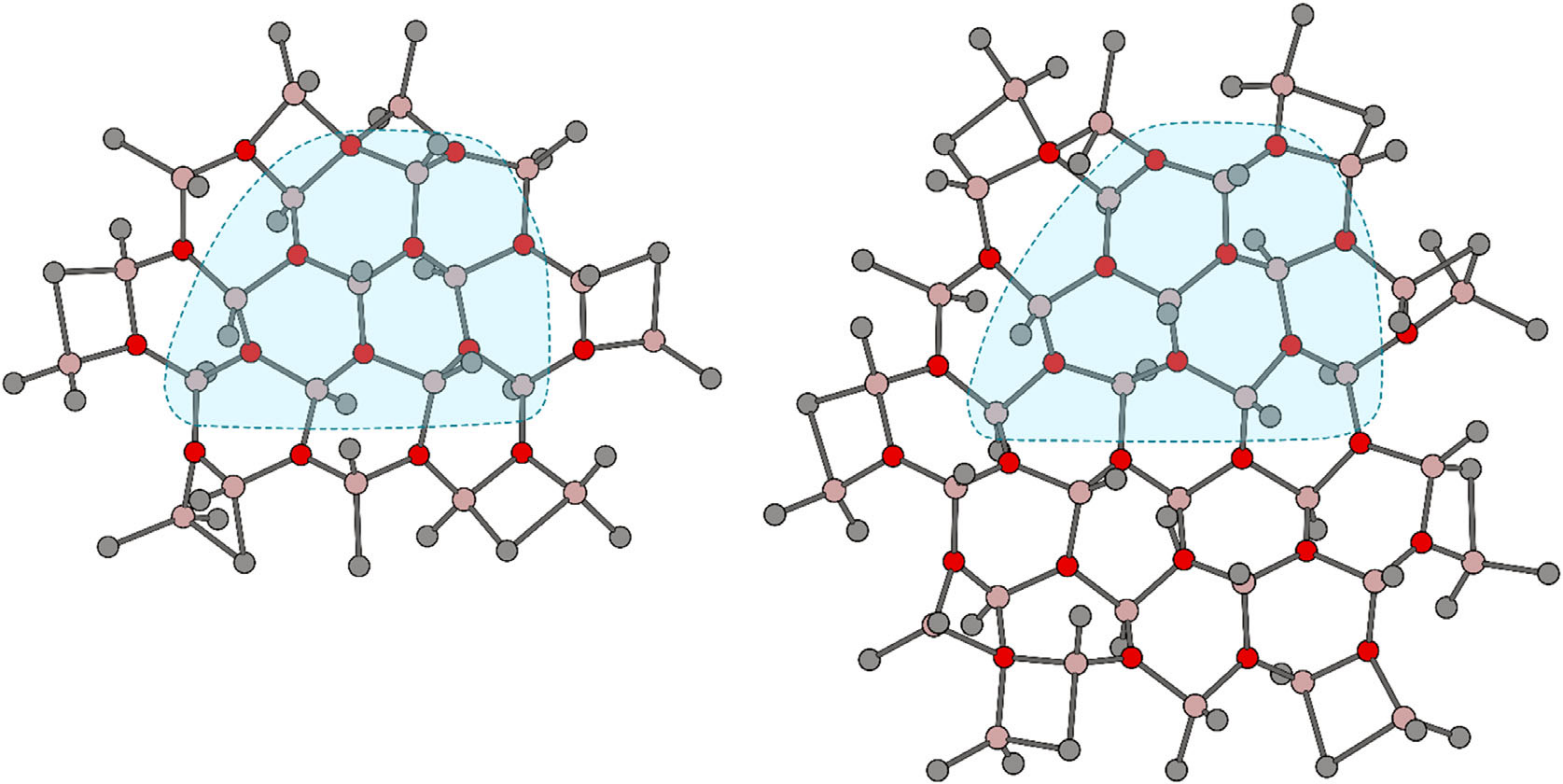
Structure of the [16,6]^−^ anion derived from the ovalene‐analogous 16,7 sheet model (left) and the crystallographically characterized 26,9 sheet (right). Areas highlighted in blue show the structural kinship between the two species. Aluminum atoms are shown in pink, oxygen in red, and carbon in gray. Hydrogen atoms are omitted for clarity.

**FIGURE 11:**
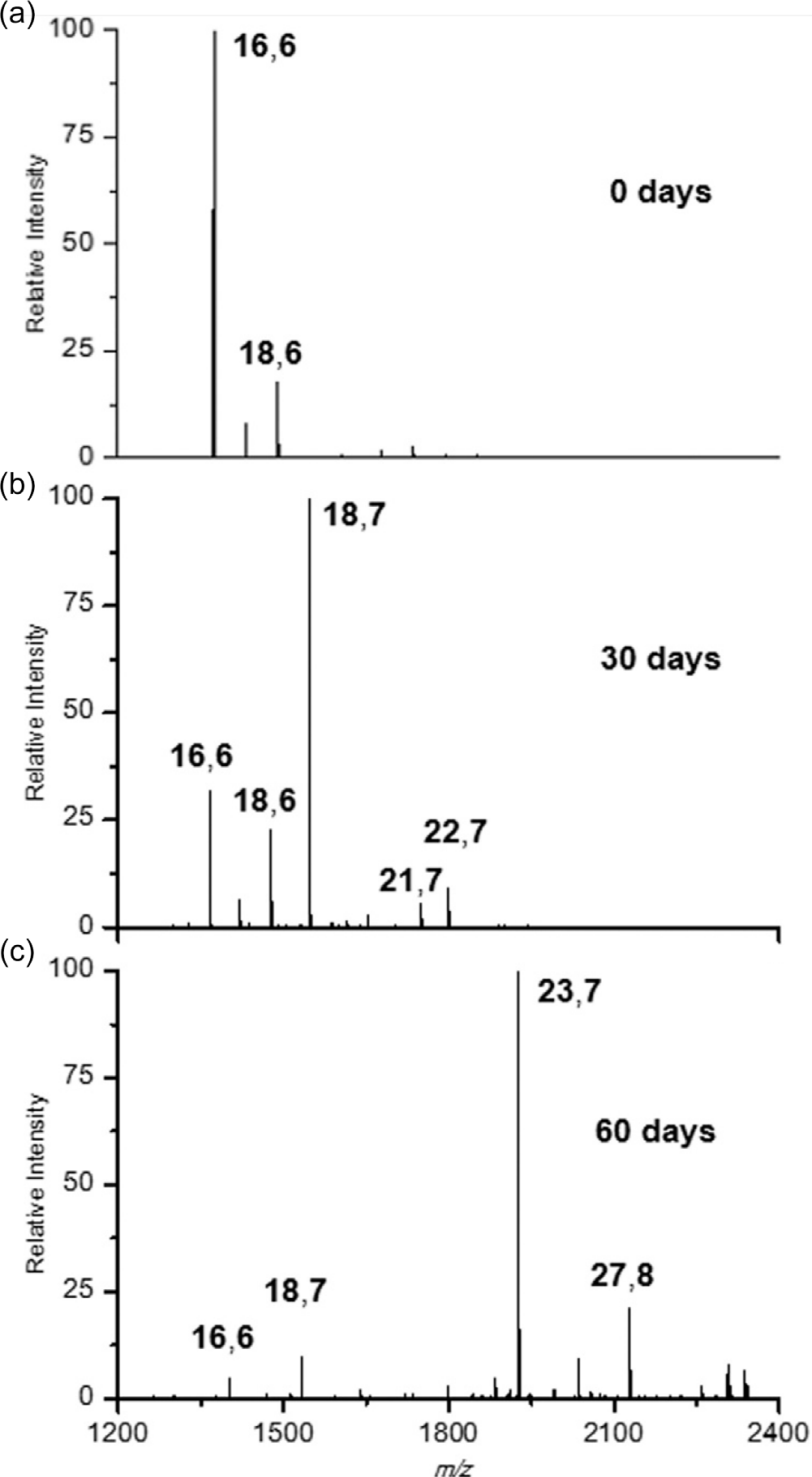
Negative‐ion ESI‐MS spectra of MAO recorded with OMTS (OMTS:Al = 1:100) at (a) 0 days, (b) 30 days, and (c) 60 days after preparation, showing the progressive shift in anion distribution from dominance of [16,6]^−^ in freshly prepared MAO toward higher molecular weight species including [23,7]^−^ upon aging at room temperature. Reproduced with permission [8]. Copyright 2017, American Chemical Society.

The energetic consequences of this structural redesign are substantial. At the highest level of theory employed (RI‐MP2 corrected free energies in toluene), the [16,6]^−^ anion derived from the ovalene‐based 16,7 precursor is more than 50 kJ mol^–1^ more stable than the [16,6]^−^ anion from the earlier hydrolysis‐derived 16,6 sheet model. This new anion finally brought theory into alignment with the experimental dominance of this species in ESI‐MS spectra. The ovalene‐based neutral 16,7 has comparable thermodynamic stability to the earlier 16,6 neutral despite incorporating one additional TMA unit. The ovalene‐based structure also shows notable geometric similarity to the crystallographically characterized 26,9 sheet isolated by Luo et al., and this structural kinship is reflected in comparable ion‐pair energetics (Figure 10) [91]. The experimental dominance of [16,6]^−^ over [26,8]^−^ in ESI‐MS spectra of commercial MAO is therefore better understood in terms of the relative abundance of their neutral precursors in solution. Direct experimental evidence for this observation comes from aging studies: freshly prepared MAO stored at low temperature is overwhelmingly dominated by [16,6]^−^, while storage at room temperature over weeks to months progressively shifts the distribution toward higher molecular weight anions such as [23,7]^−^ (Figure 11). The Sigma–Aldrich commercial material, known to have experienced more aging prior to distribution, shows this higher molecular weight distribution as its resting state [8]. The 26,9 sheet is therefore most naturally understood as a kinetic product of prolonged aging rather than a primary component of freshly synthesized MAO, and its low ESI‐MS intensity in routine measurements reflects scarcity rather than intrinsic unreactivity. With the structure of the dominant [16,6]^−^ anion now established on firm thermodynamic and experimental grounds, the question of how this species participates in catalyst activation can be addressed directly.

## 7 Catalyst Activation by MAO

A comprehensive survey of catalyst activation literature is outside the scope of this review. What motivates a focused examination here is the structural foundation now available: the ovalene‐based sheet models are the first structural proposals for MAO that are simultaneously grounded in high‐level quantum chemical calculations, consistent with ESI‐MS experimental observables, and geometrically analogous to the crystallographically characterized 26,9 sheet. It is therefore instructive to ask what these models imply for catalyst activation, and whether the discrepancy between computed and experimental insertion barriers can finally be resolved.

**FIGURE 12:**
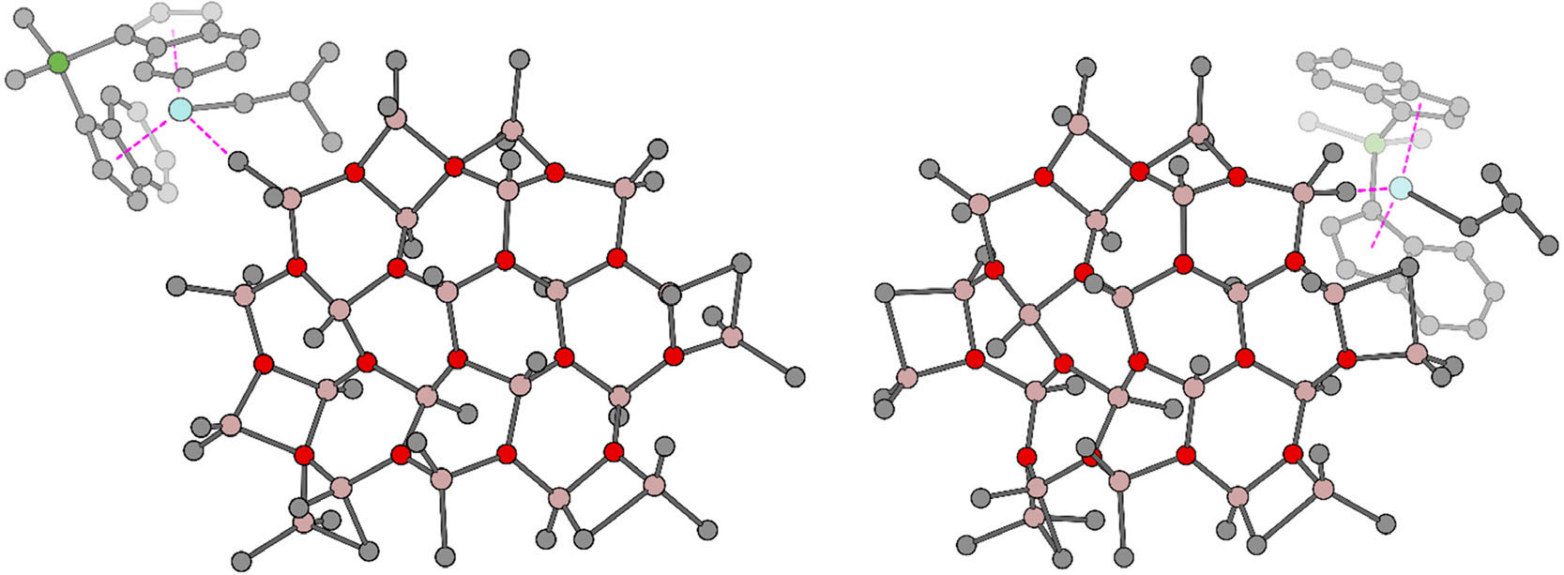
Contact‐ion pairs formed from SBIZrMe_2_ and 16,7‐O4 sheet (left) and 16,7‐O3 sheet (right), representing the two structurally distinct activation products of the ovalene‐based 16,7 precursor. The O3 isomer yields insertion barriers in agreement with experimental propagation rates, while the O4 isomer is more than two orders of magnitude less reactive at ambient temperature. Zirconium atoms are shown in blue, silicon in green, aluminum in pink, oxygen in red, and carbon in gray. Hydrogen atoms are omitted for clarity.

The question was first addressed using the earlier, hydrolysis‐derived 16,6 sheet model in combination with the well‐characterized SBIZrMe_2_ catalyst system. This system was chosen because quantitative experimental data on both activation equilibria and propene insertion barriers are available [45]. The computed insertion barrier for the contact ion‐pair formed from this model was far higher than the experimental value for MAO‐activated SBIZr and substantially higher than the barriers computed for borate‐ and borane‐activated analogs [96, 97, 98]. Insertion was found to proceed through the contact ion‐pair rather than the outer‐sphere heterodinuclear complex, which acts as a dormant state. The old sheet model thus pointed to an anion that coordinates the cation too strongly, raising rather than resolving the question of what the actual active species in MAO‐catalyzed polymerization might be. This unsatisfactory outcome was a further signal, alongside the ionization discrepancy discussed above, that the structural model itself required revision. With the ovalene‐based 16,7 neutral as precursor and [Me_2_Al]^+^ abstraction as the ionization mechanism, the picture changes qualitatively. The configurational flexibility at the edge sites of the 16,7 neutral carries over to the anion. At these edge sites, Me_2_AlO groups can coordinate either through a bridging methyl, maintaining three‐coordinate oxygen (O3 configuration), or directly through oxygen, creating four‐coordinate oxygen (O4 configuration). The anions then generate two structurally distinct contact ion‐pairs with the SBIZr cation (Figure 12) [99]. These isomers differ only in the detailed structure of the sheet counter‐anion, but their consequences for reactivity are dramatic. DLPNO‐CCSD(T)‐corrected insertion barriers differ by over 20 kJ mol^–1^ between the two, translating to more than two orders of magnitude difference in reactivity at ambient temperature. The barrier for the reactive O3 isomer is in very good agreement with the experimental propagation barrier for MAO‐activated SBIZr [98]. The origin of the difference lies in the electrostatic potential of the two anion faces: the O4 configuration presents an electron‐rich AlMe_2_ group that coordinates the zirconocenium cation strongly, locking it in a less reactive contact ion‐pair, while the O3 configuration delocalizes charge more broadly across the anion surface, allowing more facile monomer access and insertion.

**FIGURE 13:**
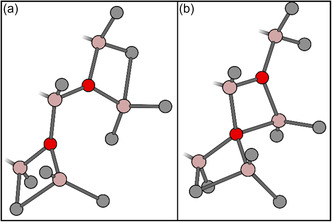
Structural differences between the (a) O3 and (b) O4 edge sites in the 16,7 sheet models. In the O3 configuration, the Me_2_AlO group coordinates through a bridging methyl, maintaining three‐coordinate oxygen; in the O4 configuration, direct coordination through oxygen creates a four‐coordinate environment. This distinction determines whether the resulting contact ion pair is catalytically active or dormant. Aluminum atoms are shown in pink, oxygen in red, and carbon in gray. Hydrogen atoms are omitted for clarity.

The two isomers thus constitute a two‐state catalyst system embedded in a single chemical composition, with the two faces of the sheet anion playing the role of a molecular switch between active and dormant states (Figure 13). Both are expected to be present in comparable amounts under polymerization conditions, since the free energy difference between the O3 and O4 configurations is modest and their interconversion has a low barrier in the free anion. Once coordinated to the zirconocenium cation, however, the two faces cannot easily interconvert, and the O4 isomer represents a kinetically trapped dormant state [99]. This framework provides a natural explanation for the well‐known observation that only a fraction of the catalyst added to MAO‐activated polymerization systems is actively growing chains at any given time [1].

The results described above pertain to a single catalyst system, SBIZr, chosen for the availability of quantitative experimental benchmarks. Whether the same two‐state picture applies across the broad range of metallocene and post‐metallocene catalysts used with MAO in practice remains to be established. The structural diversity of metallocene ligand frameworks, and the sensitivity of insertion barriers to the precise geometry of the transition state, suggest that the balance between O3 and O4 contact ion‐pairs may differ between catalyst systems, potentially contributing to the empirically observed variation in the fraction of active sites. Extending the ovalene‐based computational framework to other catalyst families and connecting the results to experimentally accessible observables such as the concentration dependence of polymerization kinetics represents a natural and important direction for future work.

## 8 Conclusions

The computational characterization of MAO has undergone a fundamental transformation over the past decade, driven by the convergence of methodological advances, systematic structural exploration, and increasingly incisive experimental validation. The central structural question that motivated this field – what is the dominant anionic component of fresh MAO? – now has a well‐founded answer, at least for the species accessible under ESI‐MS conditions. The [16,6]^−^ anion that has dominated ESI‐MS spectra of fresh commercial MAO for years possesses an ovalene‐based four‐coordinate aluminum sheet structure. This structure is preferred thermodynamically over the previously proposed cage alternatives once vibrational entropy is included in the comparison, a conclusion that holds under both harmonic and quasi‐harmonic treatments. Further, this structure is geometrically related to the crystallographically characterized 26,9 sheet, consistent with synchrotron PDF data favoring sheet models with stacking, and yields computed propene insertion barriers in excellent agreement with experiment when the configurational flexibility of the sheet edge is properly treated. The convergence of evidence from computation, mass spectrometry, NMR spectroscopy, X‐ray crystallography, and total X‐ray scattering toward a consistent structural picture represents a qualitative advance over the situation even five years ago.

The methodological lessons learned along the way are as important as the structural conclusions, and they extend well beyond MAO. The failure of M06‐2X for four‐coordinate Al–O environments, undetected because the TMA dimerization benchmark contains no oxygen, illustrates a general hazard: a functional validated on one structural motif can fail qualitatively for another that is electronically distinct. The vibrational entropy contribution proved to be the decisive thermodynamic quantity that had previously obscured the sheet‐over‐cage preference, yet its accurate computation remains challenging: the quasi‐harmonic approximation and the free volume translational entropy correction introduce errors of opposite sign that do not in general cancel, and the net effect on computed Gibbs energies for large flexible systems has not yet been fully characterized. These findings carry practical implications for computational studies of aluminum chemistry far beyond MAO: benchmarking against TMA dimerization alone is insufficient, vibrational entropy must be treated carefully for large flexible organometallic systems, and the choice of functional should be validated against the specific structural motifs present in the system under study.

The structural resolution described above pertains primarily to the dominant anionic species in fresh MAO solutions, but MAO is a complex mixture whose full structural inventory extends well beyond the reactive 16,7 sheet component. ESI‐MS, NMR, and scattering data collectively point to a broad distribution of species spanning a wide range of compositions and molecular weights, and the relationship between this distribution and the observed catalytic behavior remains incompletely understood. The active component is present at low concentration within the bulk material, and other structural types, including cage species, are expected to participate in the overall activation chemistry in ways that depend on preparation conditions, aging, catalyst identity, and aluminum‐to‐metal ratio. Understanding reactivity trends across the full structural ensemble of MAO – as a function of size, structural motif, site type, and morphology, also including the effects of aggregation in both solution and condensed phases – represents the central open challenge in the field. Meeting this challenge will require both the high‐level computational methods validated here and closer integration with experimental kinetic and spectroscopic data that can discriminate between the contributions of individual structural components. A particularly important and still unresolved aspect of this challenge concerns the treatment of the counterion and the surrounding solvent, both of which are critical to MAO behavior. The work reviewed here shows that the counterion is far from a spectator: the specific face of the sheet anion presented to the metallocenium cation determines whether the resulting ion pair is catalytically active or dormant, with insertion barriers differing by more than 20 kJ mol^–1^ between the two. Solvent effects are more difficult still, since continuum models capture only the electrostatic component of solvation while the entropic consequences of moving from gas phase to solution remain inadequately treated for systems of this size. A fully reliable computational description of counterion and solvent effects in MAO catalysis has not yet been achieved, and in our view, this represents one of the most important open challenges in the field.

Nevertheless, the structural and mechanistic foundation now available makes possible, for the first time, a genuinely rational approach to cocatalyst design in olefin polymerization. Rather than empirically varying MAO loading or preparation conditions, it is now conceivable to ask which structural features of the activator are essential for high activity, what determines the fraction of reactive sites, and how ion‐pairing geometry influences insertion barriers across different catalyst systems. The existence of MAO's molecular cousin, the homodinuclear Al‐H‐Al^+^ cation that replicates MAO's complete activation function at orders of magnitude lower aluminum loading, demonstrates that these questions have practical answers rather than merely theoretical ones. Whether the same principles can guide the design of activators with controlled Lewis acidity, defined active site concentration, and tunable ion‐pairing properties – whether based on aluminum or on other elements – is a question that the computational framework described in this review is now positioned to address seriously.

## Funding

This work was supported by the Research Council of Finland (357509); Finnish Ministry of Education and Culture (OKM/7/523/2024).

## Conflicts of Interest

The authors declare no conflicts of interest.

## Data Availability

Data sharing not applicable to this article as no datasets were generated or analyzed during the current study.
